# Genome-Wide Transcription Factor DNA Binding Sites and Gene Regulatory Networks in *Clostridium thermocellum*

**DOI:** 10.3389/fmicb.2021.695517

**Published:** 2021-09-07

**Authors:** Skyler D. Hebdon, Alida T. Gerritsen, Yi-Pei Chen, Joan G. Marcano, Katherine J. Chou

**Affiliations:** ^1^Biosciences Center, National Renewable Energy Laboratory, Golden, CO, United States; ^2^Computational Sciences Center, National Renewable Energy Laboratory, Golden, CO, United States

**Keywords:** transcriptional regulatory networks, *Clostridium thermocellum* DSM 1313, regulon, transcription factor, DNA binding site, bioinformatics

## Abstract

*Clostridium thermocellum* is a thermophilic bacterium recognized for its natural ability to effectively deconstruct cellulosic biomass. While there is a large body of studies on the genetic engineering of this bacterium and its physiology to-date, there is limited knowledge in the transcriptional regulation in this organism and thermophilic bacteria in general. The study herein is the first report of a large-scale application of DNA-affinity purification sequencing (DAP-seq) to transcription factors (TFs) from a bacterium. We applied DAP-seq to > 90 TFs in *C. thermocellum* and detected genome-wide binding sites for 11 of them. We then compiled and aligned DNA binding sequences from these TFs to deduce the primary DNA-binding sequence motifs for each TF. These binding motifs are further validated with electrophoretic mobility shift assay (EMSA) and are used to identify individual TFs’ regulatory targets in *C. thermocellum*. Our results led to the discovery of novel, uncharacterized TFs as well as homologues of previously studied TFs including RexA-, LexA-, and LacI-type TFs. We then used these data to reconstruct gene regulatory networks for the 11 TFs individually, which resulted in a global network encompassing the TFs with some interconnections. As gene regulation governs and constrains how bacteria behave, our findings shed light on the roles of TFs delineated by their regulons, and potentially provides a means to enable rational, advanced genetic engineering of *C. thermocellum* and other organisms alike toward a desired phenotype.

## Introduction

The thermophilic bacterium (55–60°C) *Clostridium thermocellum* (also known as *Ruminiclostridium thermocellum, Hungateiclostridium thermocellum* and recently renamed *Acetivibrio thermocellus*, Yutin and Galperin, 2013; Zhang et al., 2018; Tindall, 2019) has emerged as a chassis organism for consolidated bioprocessing (CBP) (Mistry and Cooney, 1989; Lynd, 1996). This process takes advantage of the bacterium’s cellulosome (Artzi et al., 2017; Yoav et al., 2017), an extracellular protein complex composed of highly orchestrated and synergistic (hemi)-cellulase enzymes to depolymerize (hemi)-cellulose (Lamed and Bayer, 1988; Felix and Ljungdahl, 1993). Solubilization and fermentation of cellulose, the most recalcitrant component of the plant cell wall, can thus be achieved in one-integrated bioreactor using *C. thermocellum* without adding separately produced cellulase enzymes (Lynd et al., 2002). The bacterium’s unique features and its industrial relevance in the conversion of biomass (lignocellulose) to a chemical or fuel draws increasing interest to understand various aspects of this microorganism. In the past decade, research related to this cellulose-degrading bacterium flourished and there are numerous studies on the bacteria’s physiology and metabolism (Xiong et al., 2016; Olson et al., 2017; Dash et al., 2019; Jacobson et al., 2020), cellulosome genesis (Yoav et al., 2017; Kahn et al., 2020), genetic tools development (Olson and Lynd, 2012; Marcano-Velazquez et al., 2019; Walker et al., 2020) genome engineering for improving the conversion of lignocellulose to a target product (Hon et al., 2017; Mazzoli et al., 2020; Tafur Rangel et al., 2020), computational modeling related to this microbe (Dash et al., 2017; Garcia et al., 2020), and beyond.

Gene transcription is one of many essential mechanisms that cells use to regulate cellular functions and respond to environmental changes to sustain life. The literature involving gene regulation in *C. thermocellum* reflects the general interest in using this thermophilic bacterium to convert biomass to biofuels (Raman et al., 2011; Wilson et al., 2013a; Wei et al., 2014), with particular emphasis on the role and regulation of the cellulosome (Mishra et al., 1991; Dror et al., 2003; Newcomb et al., 2011). The analysis of differential gene expression has also played an important part in understanding the response of *C. thermocellum* to ethanol stress (Yang et al., 2012), redox perturbation (Sander et al., 2015) and furfural and heat stress (Wilson et al., 2013b). However, these studies focus on the transcription output. Relatively few studies to date have focused on transcription factors (TFs) and their regulatory targets (i.e., regulons) in *C. thermocellum*. The three LacI-type TFs named GlyR1, GlyR2, and GlyR3 (Clo1313_2023, Clo1313_0089, and Clo1313_0396, respectively) have been characterized for their roles of regulating cellulolytic enzymes in response to sugar molecules, particularly GlyR3 (Newcomb et al., 2007; Choi et al., 2017a; Wilson et al., 2017). Sigma and anti-sigma factors have also been investigated for their role in regulating genes encoding components of the cellulosome upon sensing a specific extracellular carbohydrate (Kahel-Raifer et al., 2010; Nataf et al., 2010; Bahari et al., 2011).

Beyond a more thorough understanding of *C. thermocellum* physiology, the study of TFs, their binding sites, and their regulatory networks holds potential to unlock opportunities for advanced engineering. This is directly demonstrated by the development of a GlyR3-based regulatable gene expression system (Mearls et al., 2015). In addition, TFs have been applied to detect desirable product molecules or metabolic states (Bilan et al., 2014), select spontaneous mutants with desirable characteristics (Cheng et al., 2018), control metabolic pathway expression levels for improved product (Xu et al., 2014), and more (Mahr and Frunzke, 2016; Cheng et al., 2018). On the other hand, TF binding DNA sequences have been used as decoys to titrate TFs away from influencing their native target genes and thereby modulate metabolic activities in *E. coli* (Wang et al., 2021). We see ample opportunity for advances in both basic understanding and applied engineering of TFs and gene regulation in *C. thermocellum.*

Methods for determining the role of a TF can be categorized across various spectra: from low- to high-throughput, from single-target to whole-genome, from experimental to bioinformatic, from detecting the causal TF-DNA interaction to detecting the effect on transcription. These methods are previously reviewed (Stormo and Zhao, 2010; Dey et al., 2012; Furey, 2012; Rajeev et al., 2020). For this study, we chose to use DNA-affinity purification and sequencing (DAP-seq) to identify genome-wide DNA binding sites for numerous TFs in parallel (O’Malley et al., 2016; Bartlett et al., 2017). DAP-seq is analogous to ChIP-seq in that both methods identify enriched genomic DNA fragments from TF-DNA complexes using next-generation sequencing. These methods diverge in how the TF-DNA complexes are obtained. Instead of using immunoprecipitation to purify TF-DNA complexes from cells, DAP-seq mixes pre-purified TFs and genomic DNA fragments. The use of magnetic beads to pull down affinity-tagged TFs expressed via *in vitro* transcription and translation (IVTT) facilitates high-throughput selection of TF-DNA complexes. DNA fragments released from heat-denatured TFs are then barcoded and amplified for multiplexed next-generation sequencing. The advantages of using IVTT and an *in vitro* environment for TF-DNA binding come with the costs of potentially producing DNA-binding-incompetent TFs due to protein misfolding or the absence of the essential cofactors or effector molecules necessary to induce a DNA-binding TF conformation. DAP-seq was first developed using *Arabidopsis* TFs (O’Malley et al., 2016; Bartlett et al., 2017) and was recently applied to *Pseudomonas putida* (Trouillon et al., 2020, 2021).

In this study, we screened for genome-wide DNA binding sites for nearly one hundred *C. thermocellum* TFs using DAP-seq. Upon identification of TF-DNA binding sequences, we integrate findings from bioinformatics (e.g., discovery of binding sequence motifs), experimental validation, and prior knowledge of the TFs’ homologues along with their regulatory targets to reconstruct a gene regulatory network in *C. thermocellum*. This network encompasses TFs and respective regulons (sub-networks) with previously characterized homologues (such as LexA Clo1313_1449 and Rex Clo1313_2471) as well as those not previously known. In this reconstructed gene network, there are regulons as small as a two-gene operon and as large as nearly 100 genes in 83 operons. To our knowledge, this study is the first large scale DAP-seq analysis of bacterial TFs and adds substantially to the body of work describing transcriptional regulations in *Clostridium thermocellum*.

## Materials and Methods

### Strain and Culturing

*Acetivibrio thermocellus* and *Ruminiclostridium thermocellum* also known as *Hungateiclostridium thermocellum* which is commonly known and referred to herein as *Clostridium thermocellum* DSM 1313 (Yutin and Galperin, 2013; Zhang et al., 2018; Tindall, 2019) is used as the host for this study. Unless otherwise noted, the bacteria are cultured routinely in 55°C, CTFUD rich medium (Olson and Lynd, 2012) and strict anaerobic condition supplemented with 5 g/L of cellobiose. The culture species purity is validated regularly with PCR using a primer pair (5′-GCCAAGGCATCCACC-3′ and 5′-GTCGTAACAAGGTAGCCGTA-3′) which generates unique PCR products for DSM 1313 (Cardinale et al., 2004).

### Identifying Putative TFs in *C. thermocellum*

Putative transcription factors were identified and compared using various search functions across several databases. We searched the *Hungateiclostridium thermocellum* LQ8, DSM 1313 genome housed in the Department of Energy’s (DOE’s) Joint Genome Institute Integrated Microbial Genomes and Microbiomes (Nordberg et al., 2014; Chen et al., 2019) and the *Ruminiclostridium thermocellum* DSM1313 genome in the BioCyc database (Karp et al., 2019) for protein-coding genes with functional (COG, KOG, pfam) annotations relevant to transcriptional regulation. We also used BLAST2GO (Gotz et al., 2008) to aid our search. Precompiled lists of TFs for many prokaryotes are available on the Predicted Prokaryotic Transcription Factor database (p2tf.org, Ortet et al., 2012).

### Preparation of Genomic DNA Libraries for DAP-Seq

Total genomic DNA (gDNA) was extracted from fresh overnight cultures of *C. thermocellum* when optical density at 600 nm reached around 1.2 using the DNeasy Blood and Tissue kit (Qiagen cat. no. 69506) and following the protocol for gram-positive bacteria. The gDNA concentration was measured with the Qubit 3.0 Fluorometer (Thermo Fisher, cat. no. Q33227). A total of 33 μg of gDNA was sent to the University of Idaho Genomics Resources Core for the preparation of a DAP-seq-ready DNA library. At the facility, the low molecular weight fragments of 50–100 bp were removed using a DNA bead purification protocol (Ampure XP, cat. No. A63880). The gDNA was then sheared to ∼200 base pair fragments using a S-series focused ultrasonicator (Covaris, cat. no. 500295). Primer adapters were ligated to the ends of the 200 bp fragments generated by the sonication step. The efficiency of the ligation steps of the adapters to our gDNA fragments was measured using quantitative PCR (qPCR, Kapa Biosystems, cat. no. KK4828) indicating that approximately 25% by weight of the gDNA fragments were successfully ligated at both ends, yielding ∼8 μg of adapter-ligated DNA.

The qPCR reaction was performed using primers that contain the Illumina adapter sequence (p5 and p7) as well as a unique index sequence (barcode) designed for multiplexing the sequencing reaction step. An example of these primers is: i5_duplex_primer_1, 5′-*AATGATACGGCGACCACCGA*G ATCTACACNNNNNNNNACACTCTTTCCCTACACGAC-3′; and i7_duplex_primer_1, CAAGCAGAAGACGGCATACG AGATNNNNNNNNGTGACTGGAGTTCAGACGTGT. The “NNNNNNNN” region of these primers represent the unique DNA indices (barcodes), the underlined sequence represents the p5 (i5) and p7 (i7) Illumina sequences.

### Cloning and *in vitro* Transcription and Translation (IVTT) of Putative TFs

Genes encoding putative TFs were codon optimized for expression in *E. coli* and cloned by GENEWIZ into pFC20A (Promega, cat. no. G1681) at the SgfI and XhoI cut sites. The final constructs encode TFs with a C-terminal HaloTag. We performed DAP-seq assays following published protocols (Bartlett et al., 2017) with a few modifications tailored for TFs from a thermophilic and anaerobic bacterium. The *E. coli* T7 S30 Extract System for Circular DNA (Promega, cat. no L1130) was used for IVTT of putative TFs according to the manufacturer’s recommendation. For a large-scale assay of 131 putative TFs in triplicates, a total reaction volume of 150 μl and 3 μg of the plasmid DNA template was initially setup and subsequently split into three DAP-seq reactions. The IVTT reactions were performed anaerobically at 37°C for 4 h. The TF-HaloTag fusions were loaded onto Magne^®^ HaloTag^®^ Beads (Promega) while nutating for 1 h at room temperature under anaerobic conditions. During this time, TF proteins with HaloTags formed covalent attachments to the magnetic beads. The TF-bound beads were then washed with PBS+NP40 solution three times to discard untagged, non-specific proteins and resuspended in 120 μl of PBS+NP40. The magnetic beads with covalently bound TFs were split into three wells in a 96-well PCR plate. We also performed small-scale DAP-seq assays involving few targeted TFs and conditions. Five technical replicas were setup for each TF in the small-scale DAP-seq and reaction volumes were proportionally scaled for five parallel replicates. A negative control plasmid without a TF was also constructed in such a way that the TF amino acid sequence was replaced with the amino acid sequence MHHH, resulting in a construct that encodes a MHHH-HaloTag fusion. Whether the experiment is setup to be small- or large-scale, an IVTT without expressing a TF is performed to provide a negative control for downstream assays.

### DAP-Seq DNA Pull-Down

TF-binding gDNA fragments were pulled-down by DAP-seq assays. We added 40 μL of Qiagen elution buffer containing 12.5 ng of gDNA library to TFs that were previously attached to magnetic beads. The TFs and DNA were incubated at 55°C for 2 h under anaerobic conditions while nutating. Free DNA was separated from bead-bound TF-DNA complexes by repeatedly washing the magnetic beads with fresh PBS+NP40. TF-bound DNA was recovered for analysis by denaturing the TFs at 98°C for 10 min. PCR was then performed to simultaneously amplify the adapter-ligated DNA and add barcoded adapters to facilitate multiplexed sequencing as previously described (Bartlett et al., 2017). Five microliters of each PCR reaction were pooled together, and 238 μL of the pooled samples were separated on a 1% agarose gel. DNA fragments between 200 and 400 bp were purified from the gel and eluted in Qiagen elution buffer. This multiplexed library of affinity-purified DNA fragments was sequenced using a 2x150 bp paired-end strategy on an Illumina HiSeq 4000 instrument.

Two sets of DAP-seq experiments were performed in addition to the large-scale assay of 131 putative TFs. Eight TFs were re-assayed in the absence and presence of various amounts of the soluble fraction of cell lysate to test if co-factors in the lysate would improve TF-DNA binding and therefore detection of binding sites by DAP-seq (because the TFs may change their protein conformation upon binding to an effector). Finally, 13 TFs were assayed in five parallel replicates to assess reproducibility of the assay, and a new DNA library was freshly prepared for this set of DAP-seq experiment. Each assay included the MHHH-HaloTag construct negative control in addition to the TFs.

### DAP-Seq Data Analysis

The reads output from sequencing DNA fragments from purified TF-DNA complexes were assessed for quality using custom software packages^1^ and were of overall high quality (above Q-30). Reads were mapped to the *Clostridium thermocellum* DSM 1313 genome (RefSeq Accession NC_017304) using bowtie2 (Langmead and Salzberg, 2012). Regions on the genome with greater local depth of mapped sequencing reads were identified using GEM and are hereafter referred to as “DAP-seq peaks” (Guo et al., 2012). Two parameters were modified from the defaults: putative duplicate read elimination was turned off; and the threshold sequencing depth relative to the negative control for peak calling was set to one. Results were output in the BED file format for input into Bedtools 2.28^2^ for peak and gene annotations using the “annotate” function in conjunction with the *C. thermocellum* GFF file. Annotated peaks were then sorted by position using the sort-bed utility in BEDtools. Sorted peaks were input into the bedmap utility from the BEDOPS 2.4.39 program^3^ to calculate statistics and overlaps for all the putative annotations. This analysis resulted in a list of peaks for each set of parallel, replicate DAP-seq experiments performed.

### Motif Discovery

We analyzed the DNA sequences of the DAP-seq peaks for each TF, both on a per-experiment basis and collectively, to identify enriched sequence motifs using tools from MEME-Suite 5.1.1 (Bailey et al., 2009). By the nature of DAP-seq experiments, sequencing read depth is expected to be the highest at TF binding loci. We identified sequence motifs enriched at the apex of 500 bp wide peak sequences relative to the *C. thermocellum* genome using MEME-ChIP (Machanick and Bailey, 2011). An important aspect of TF-DNA binding motif discovery is the identification of many similar binding sites embedded among many unique genomic contexts. Having long stretches of duplicate or highly similar sequences can interfere with motif discovery. We reduced this interference while still preserving the variety of sequences by using the purge utility with a cut off score of 1,600. For sequences of 500 nucleotides this removed duplicate peaks and repeats such as transposon elements that are larger than TF binding sites. MEME-ChIP parameters were set to find motifs between three and 60 nucleotides in length using a second-order Markov model of the *C. thermocellum* genome as a background model. Other parameters were left at default (Machanick and Bailey, 2011). Files containing sequences before or after purging or sites contributing to the final motif are available in a Supplementary Folder.

We compared these motifs to those of published TFs using TOMTOM, which is also part of the MEME-Suite (Gupta et al., 2007). We searched the combined prokaryotic motif databases using a threshold of *q* < 0.05.

### Protein Purification and EMSAs

Electrophoretic mobility shift assays (EMSAs) were performed to confirm TF-DNA binding sequence motifs identified by DAP-seq data analyses. The C-terminal HaloTag coding sequence was replaced with a 6xHis tag by amplifying the TF-encoding pFC20A plasmids with Long-Amp Taq DNA polymerase using primers prSDH_051 (AGTGATGGTGATGGTGATGGGATCCATCG TTATCGCTCTGAAAGTA) and prSDH_052 (TGGATC CCATCACCATCACCATCACTAATAGAATTCTAGAGTCGAC CTGCAGG). These primers introduce KpnI cut sites allowing the PCR-linearized vector to be closed by restriction digest and ligation. The accuracy of the DNA sequences in these constructs were validated by commercial Sanger sequencing (GENEWIZ).

His-tagged TFs were expressed in *E. coli* BL21 DE3 cells. One-liter batches of LB supplemented with ampicillin (100 μg/ml) were inoculated with 10 mL of overnight cultures. Protein production was induced with 1 mL of 0.5 M IPTG when the culture reached an optical density of approximately 0.8. Induced protein production continued overnight at room temperature. Cells were harvested by centrifugation, resuspended in ∼30 mL of PBS with 25 mM imidazole and lysed on ice using a QSonica Q500 sonicator with a CL-334 probe for 5 min on a 50% duty cycle at 30% power. Cell debris was pelleted by centrifugation at 27,000 relative centrifugal units (×*g*) for 55 min at 4°C. The supernatant was incubated with 1 mL of pre-equilibrated HisPur nickel-NTA resin (Thermo Scientific, cat. no. 88225) in a rocking ice-water bath for 30 min. The resin was collected by passing all the supernatant through the accompanying spin column with repeated centrifugation at 600 × *g* for 2 min. The resin was washed and spun five times with 5 mL of PBS containing 25 mM imidazole. Proteins were eluted by washing three times with 1 mL of PBS containing 250 mM imidazole. The eluent was pooled and concentrated using a 0.5 mL, 10 kDa-cut-off Amicon concentrator and buffer-exchanged with PBS containing NP40. Total protein concentration was measured using BioRad protein quantification assay with BSA as the standard. The proteins were kept at 4°C or on ice to reduce precipitation.

EMSAs were performed with 0.5 μM double-stranded DNA oligo probes (IDT DNA) and various amounts of semi-pure TFs. Probes were synthesized to match the best (lowest *p*-value) site in the MEME-ChIP aligned peak sequences. The probe sequences include a representative sequence of the motif plus 10 bp on each flank in context of the peak from which it was derived. Probes without matches to the expected motif (*p* > 10^–3^) were used as negative controls. After combining the probe and protein, the volume was brought to 10 μL using PBS+NP40, and 3 μL of 5x sucrose-based loading buffer was added. Samples were loaded onto 10-well, 6% polyacrylamide DNA retardation gels (Invitrogen, cat. no. EC6365BOX) and subjected to 100 V for 1 h with room temperature 0.5× TBE (Invitrogen, cat. no. LC6675) as the running buffer without temperature control. Gels were stained in 50 mL of running buffer with 1× for 20 min while rocking and imaged on a BioRad GelDoc. EMSA images were quantified using ImageJ (Schneider et al., 2012). We chose to not calculate affinity constants for the TF-DNA interactions because the TFs were not purified to homogeneity.

### Identification of Putative TF Binding Sites

The DNA binding sequence motifs inferred by MEME-ChIP were used to identify TF binding positions in peak sequences and in non-coding regions of the genome. Matrix-based scanning protocols within Regulatory Sequence Analysis Tools (RSAT) were used to estimate the probability that a given peak sequence belongs to the set of sequences encompassed in a position-specific scoring matrix (PSSM) with a threshold of *p* < 10^–4^ (Turatsinze et al., 2008). The PSSMs that we used are quantitative representations of the sequence logos discussed below. Non-coding sequences from up to 400 bp upstream to 40 bp into the coding sequence of all genes were also scanned for potential binding sites. Threshold parameters for genome scanning were selected based on negative control scanning experiments where the sequences or xrmotifs were randomized prior to analysis. A second-order Markov model of the *C. thermocellum* genome was used as a background reference for all scans.

### Comparative Genomics Searches for Conserved Regulatory Target Genes and Functions

We also evaluated whether the putative TF DNA-binding sites identified above were evolutionarily conserved. The non-coding sequences upstream of genes with either a predicted binding site (*p* < 10^–5^) or a DAP-seq peak, along with the corresponding region from similar genes in related organisms, were scanned for conserved binding sites using RSATs footprint-scan function (Nguyen et al., 2018). Every gene in the downstream operon (or operons for the case of divergently transcribed operons), was tested independently to account for potential differences in operon structures between organisms. The genomes used in this search are listed in Supplementary Table 1. This set selects thermophilic Clostridia, particularly from the genera *Thermoanaerobacter* and *Caldicellulosiruptor*, for their potential industrial application similar to *C. thermocellum*.

The biological role of a TF is often characterized by the collective functions of its target genes. We tested if gene functions were enriched among genes with high scoring matches to the motif using GOMo (Buske et al., 2010). Such analyses are generally improved when multiple related genomes containing similar TFs are included. Genomes containing bidirectional best BLAST hits of each TF are shown in Supplementary Table 1. Gene ontology (GO) annotations were assigned to protein coding genes for all genomes. We used GO terms from the UniProt Gene Ontology Annotation Database (version goa_uniprot_all.gpa.196.gz). When annotations were not available for a genome, we used GO terms from equivalent proteins identified using DIAMOND, an efficient alternative to BLAST (Buchfink et al., 2015). GO annotations were also taken from the *Clostridium thermocellum* DSM 1313 genome files on the RSAT Teaching server (genome version 2017-05-01.072608).^4^ Default GOMo parameters were used except negative controls were derived from 10,000 sets of shuffled scores.

### Visualization of Gene Regulatory Networks

Networks of TFs and their putative target genes were visualized using Cytoscape 3.8.0 (Franz et al., 2016). Edges connecting TF nodes to their putative target gene nodes contain a summary of which experiments identified the target (large-scale and/or targeted DAP-seq, genome scanning and/or comparative genomics approaches) and a description of the positions and statistical values for peaks and binding sites. The networks for all TFs are shown in Supplementary File 1 and the raw data supporting the inclusion of each target gene in the network are listed in Supplementary Table 2.

## Results and Discussion

**FIGURE 1:**
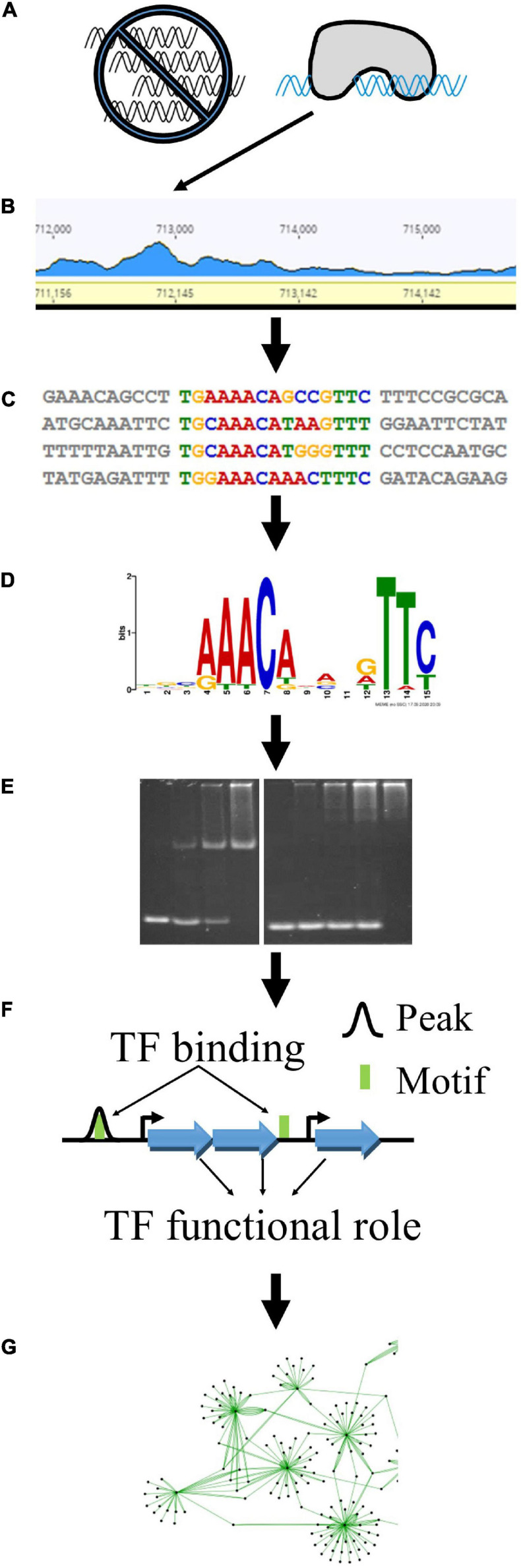
Experimental and bioinformatic approach to determine TF regulatory networks. This approach begins with large-scale analysis of putative TFs by DAP-seq. TF-bound DNA fragments are purified away from a genomic DNA library **(A)** and identified as local peaks in the mapped sequencing read landscape **(B)**. The sequences underlying these peaks **(C)** are further analyzed to find enriched DNA sequence motifs **(D)**. We validate the interaction between TFs and their respective motifs using EMSAs **(E)**. Finally, we use the locations of DAP-seq peaks (black curves) and additional TF binding sites (green boxes) to identify downstream target genes (blue arrows) **(F)** that comprise the regulatory networks **(G)** of these TFs.

Control over gene transcription is one of several layers of regulations that govern essential biological functions. We used a combination of complementary experimental and bioinformatic techniques to characterize TFs from *Clostridium thermocellum*. The core progression of our approach is depicted in Figure 1. We first used DAP-seq to identify genomic loci that were enriched among purified TF-DNA complexes (Figure 1A). These regions of higher depth of reads mapped against the genome led to experimentally identified “peaks” and are referred to as “DAP-seq peaks” hereafter (Figure 1B). From the sequences underlying those peaks (Figure 1C), we deduced TF-binding DNA sequence motifs (Figure 1D) which we validated using EMSAs (Figure 1E). We then applied bioinformatics methods to search for the experimental, DAP-seq derived motifs across the entire genome to ensure that all TF-DNA binding sites were captured, as some may not have been uncovered by DAP-seq owing to potential experimental bias. Based on the proximity of the DNA sequence motif to the nearby downstream gene-coding sequences, we identified the TF’s putative regulatory target genes and thereby gained insights to the TF functions (Figure 1F). Using these results, we constructed gene regulatory networks for these TFs (Figure 1G).

### DAP-Seq Results and Peak Identification

We initially identified and assayed 131 putative TFs (listed in Supplementary Table 3) using DAP-seq (*n* = 3 for each TF). Sequencing of multiplexed DAP-seq libraries yielded approximately 350 million paired-end, 150 bp-long reads, resulting in an overall genome sequencing depth of about 75X. The preferential binding of TFs resulted in an overabundance of reads at specific loci, or DAP-seq peaks. Ten TFs yielded peaks ranging from 1.1 to 12.2 times the local depth of mapped reads relative to the negative control, and the TFs that resulted in TF-DNA binding events are summarized in Table 1. We will refer to these TFs by their family name and abbreviated locus tag unless they have been previously named. Peak counts per TF (Table 2) ranged from 34 peaks for BlaI _0696 to 810 for LexA_1449. A list of all peaks per TF, the peaks’ genomic positions, fold-changes in depth for the peak and its associated significance (*Q*-value), the nearest ORF and the peak’s relative position to the ORF is in Supplementary Table 4.

**TABLE 1:** TFs with potential binding sites identified by DAP-seq.

TF locus tag	TF family	TF annotation
Clo1313_0026	Xre	Transcriptional regulator
Clo1313_0089^a^	LacI	GlyR2, lac repressor
Clo1313_0222	AraC	Putative protein
Clo1313_0692	TetR	Tetracycline resistance repressor
Clo1313_0696	BlaI	Beta lactamase repressor
Clo1313_1449	LexA	SOS response repressor
Clo1313_1482	GntR	Gluconate operon repressor
Clo1313_1691	Fur	Ferric uptake regulator
Clo1313_1845	BlaI	Beta lactamase repressor
Clo1313_2225	Xre	TAS II antitoxin
Clo1313_2471	Rex	Redox sensitive repressor

*^a^Binding sites for GlyR2 (Clo1313_0089) were found only in targeted repeat DAP-seq experiments, not the large-scale screen.*

**TABLE 2:** Peak counts and correlation of peak fold changes between the original, large-scale screen and a targeted DAP-seq repeat experiment of a few selected TFs.

TF	Original	Repeat	Overlap^a^	r^b^
Xre_0026	596	11	9	0.10
AraC_0222	234	322	201	0.51
TetR_0692	100	316	42	−0.13
BlaI_0696	34	22	20	0.70
LexA_1449	810	47	41	0.41
GntR_1482	407	17	14	0.55
Fur_1691	217	386	205	0.65
BlaI_1845	122	8	5	−0.35
Xre_2225	65	0	–	–
Rex_2471	128	4	3	0.18

*^*a*^The number of 200 nucleotide-wide peaks from the original experiment that overlap a peak from the repeat experiment.*

*^*b*^Pearson coefficient of peak fold changes across experiments. A fold change of zero was assigned if no overlapping peak was found.*

In a later DAP-seq experiment, we selected nine potentially cofactor- or effector-dependent TFs (Supplementary Table 5) and repeated DAP-seq experiments (using the original library and proteins freshly expressed by IVTT) in the absence and presence of varying amounts of cell lysate to test for improved peak identification (*n* = 5 for each condition). The cell lysate is presumed to provide cellular components including cofactors, metabolites, and other proteins which may be required to trigger TF-DNA binding. In these assays, we found peaks for a TF (GlyR2, Clo1313_0089) for which no peaks were identified in the previous DAP-seq assay. However, similar counts of peaks were identified whether lysate or water was added. Consistent to the first DAP-seq experiment, a few peaks were found for Rex_2471, but the addition of lysate had no appreciable affect (data not shown). These peaks for Rex were not a significant addition to the 128 previously identified peaks. Peaks for both proteins are listed in Supplementary Table 4. With these TFs and conditions, we did not see any appreciable effect of adding crude cellular extracts to the DAP-seq assay.

To test the reproducibility of the resulting peaks, we repeated the DAP-seq assay (with freshly prepped DNA library and proteins) for the 10 TFs for which peaks were found in the original screen but with five replicates each. The relative sequencing depth at the best peak remained within range of the original assay, 13.8 vs. 12.2. These peaks are also listed in Supplementary Table 4. The number of peaks identified changed substantially for all TFs except BlaI _0696. For six of the TFs, about 5% or less of the peaks in the original experiment overlapped peaks from the repeat experiment (Table 2). For the remaining TFs, a larger count of peaks was shared between the two experiments, and the total number of peaks increased by nearly 100 or more. We also quantified the correlation between peak fold changes of the two experiments (Table 2). Again, BlaI_0696 showed the best correlation with a Pearson coefficient of 0.7. Most were around 0.5 or below (Table 2) indicating poor correlation (Supplementary Figure 1).

#### Evaluation of and Potential Improvements for Our DAP-Seq Assays

In all, peaks were identified for 11 TFs, 10 during the large-scale assay. After the assays were performed, we discovered that 40 of the 131 putative TFs assayed did not have a recognizable DNA binding domain or are expected to bind DNA without sequence specificity based on the domains detected by the conserved domain search at NCBI (Marchler-Bauer et al., 2015, 2017). These proteins would not be expected to produce peaks in a DAP-seq assay. Excluding these from the total, the peak-discovery rate for the large-scale assay is around 11% (10 out of 91).

On the surface, the 11% discovery rate and poor correlation between sets of experiments with the same TF are in contrast with the 30% discovery rate and high repeatability of a DAP-seq study of *Arabidopsis* transcription factors. In the *Arabidopsis* study, bZip- and NAC-family proteins showed cross-experiment correlations with Pearson coefficients between 0.71 and 0.99 (O’Malley et al., 2016). However, it was noted that those results were heavily influenced by the protein family to which each TF belongs. The NAC and bZip TFs produced peaks up to 1,000-fold higher than background with best correlation occurring for peaks above 10-fold (Supplementary Figure 1B in O’Malley et al., 2016). Most peaks for the *C. thermocellum* TFs were less than 10-fold. This may suggest that the observed low reproducibility of peaks may be the norm for peaks at this range of fold change, and users should target a higher range of fold change in sequencing depth. Depth of sequencing and reproducibility of peaks has been shown to improve with increased amounts of genomic DNA incubated with the TFs (O’Malley et al., 2016; Bartlett et al., 2017). In line with this trend, a study of Xre-type TFs from *Pseudomonas putida* showed greater enrichment of DAP-seq peaks than our study, possibly because they used about 2.5 times as many genomic DNA copy equivalents as in our initial screen (Trouillon et al., 2021). It is also worth noting that 10 of the 11 TFs that produced DAP-seq peaks in our screen are from protein families that generally exhibit repressor functions. A good success rate was also seen for the Xre TFs from *P. putida*, with peaks being identified for seven of the eight repressors that were tested (Trouillon et al., 2021). This could be because a DNA-binding-competent state is often the default for repressor proteins. Activators, on the other hand, may require a ligand or co-activator to shift to a DNA-binding state. DAP-seq could be a more favorable assay for transcription repressors and constitutive activators than for conditional activators that require ligands for DNA binding. A step to confirm expression of all TFs via IVTT could potentially deconvolute the contributions of poor protein expression and TF inactivity due to missing effector molecules. DAP-seq could potentially be performed using only the DNA binding domain of a TF to avoid interference caused by regulatory or effector domains. Overall, we expect that using higher concentrations of genomic DNA and robust expression of full-length TFs or just their DNA binding domains can improve future assays.

### Identification of Putative DNA Binding Motifs

**FIGURE 2:**
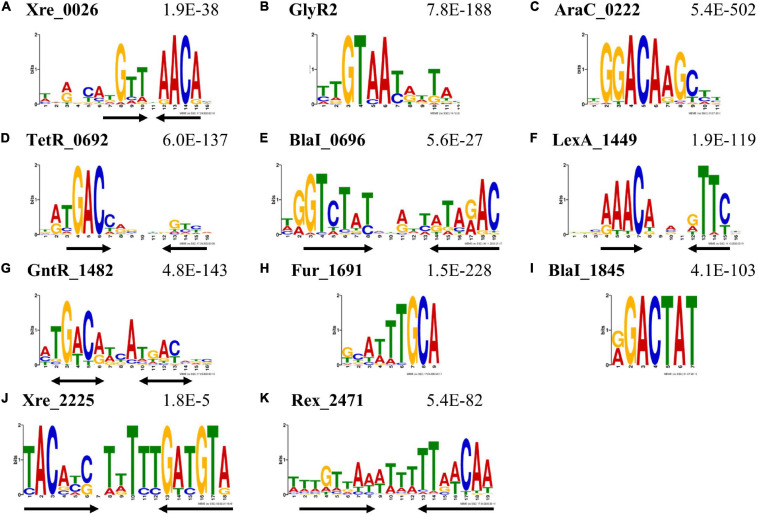
DNA sequence motifs enriched among DAP-seq peak sequences. These sequence logos represent the putative TF-DNA binding motifs derived from DAP-seq peak sequences using MEME-ChIP. The locus tag of the TF and the *E*-value estimating the frequency of finding such a motif by random chance are listed above each sequence logo. Black arrows beneath the logos indicate the location and orientation of symmetric elements. Motifs were found for TFs of various types: **(A)** Xre _0026; **(B)** GlyR2; **(C)** Xre_0222; **(D)** TetR _0692; **(E)** BlaI _0696; **(F)** LexA _1449; **(G)** GntR _1482; **(H)** Fur _1691; **(I)** BlaI _1845; **(J)** Xre_2225; and **(K)** Rex _2471. The black double arrows **(G)** indicate potentially palindromic sequences. See the Supplementary Material for input files and raw sequences contributing to these motifs.

TFs regulate gene transcription by binding to DNA in a way that inhibits or activates RNA polymerase activity at that gene or operon. Each DAP-seq peak ideally represents a TF binding event and a position for potential interaction with RNA polymerase. As sequencing reads are expected to overlap the most and have the highest relative local sequencing depth at TF binding sites, we used MEME-ChIP to search for DNA sequence patterns, or motifs, that are enriched near the apex of DAP-seq peaks relative to the *C. thermocellum* genome. A sequence motif was identified among peaks from each experiment for each TF (Figure 2). The primary features of the motif for a given TF were preserved across all DAP-seq experiments, so far as peaks were detected (Supplementary Figure 2), which overcame the limitations due to the poor reproducibility of the DAP-seq peaks. Furthermore, motifs derived from the collection of peaks from all experiments per TF matched their respective motifs from individual experiments (Supplementary Figure 2).

A sequence logo depicting a representative motif for each TF is shown in Figure 2. Seven of these motifs show dyad symmetry (either inverted or direct repeats, depicted as black arrows) indicative of two protein monomers binding in close proximity or in a dimeric conformation (Figure 2). The motif for GntR_1482 has two nearly palindromic repeats marked by bidirectional arrows (Figure 2G). In these sequence logos, the height of a letter correlates to the probability of finding the same nucleotide at that position in the motif. *E*-values estimate the number of motifs with similar characteristics that are expected to be found among sequences with randomly repositioned nucleotides and are shown in Figure 2. The Xre_2225 motif (Figure 2J) has the highest (least enriched) *E*-value of 1.8e-5; *E*-values for all other TFs are below 1e-26. These values indicate that the sequence motifs are highly enriched among their respective peaks. The representative motifs depicted in Figure 2 are either the most enriched (lowest *E*-value) MEME-ChIP motif in the collective set of peaks or an equivalent motif derived from a single experiment that shows higher-order symmetry, such as an inverted repeat. The only exception is the motif reported for Xre_2225 (Figure 2J). The motif originally identified for Xre_2225 was present only among peaks with low fold change and conspicuously absent from among peaks with the highest fold change. We reanalyzed the sequences excluding low fold change peaks and found the motif shown in Figure 2J to be present in the sequences of higher fold change peaks. The motif shown in Figure 2J is thus a resulting motif after we filtered for DAP-seq peaks for Xre_2225 with a fold-change of at least two. There was no change in the motifs of other TFs when we analyzed peaks with enrichment of twofold or higher.

We compared these motifs to those available in the MEME-Suite databases for prokaryotes (Gupta et al., 2007). The motifs for GlyR2 (Clo1313_0089), TetR_0692 and LexA_1449 (Figures 2B,D,F) share significant similarity (*q* < 0.05) with the published DNA binding motifs from TFs with similar functions. The motif for Rex _2471 is also similar to the motif of RexA from *Clostridium acetobutylicium* though it is not present in these databases (Zhang et al., 2014). These findings are evidence that the DAP-seq-derived motifs represent the DNA binding sequences for their respective TFs. Matches were found for most other motifs using default thresholds (*q* < 0.5), except for BlaI_1845 and Xre_2225. These thresholds are excessively inclusive, and therefore these two motifs appear to be unique to this study.

### *In vitro* Confirmation of TF-DNA Binding

**FIGURE 3:**
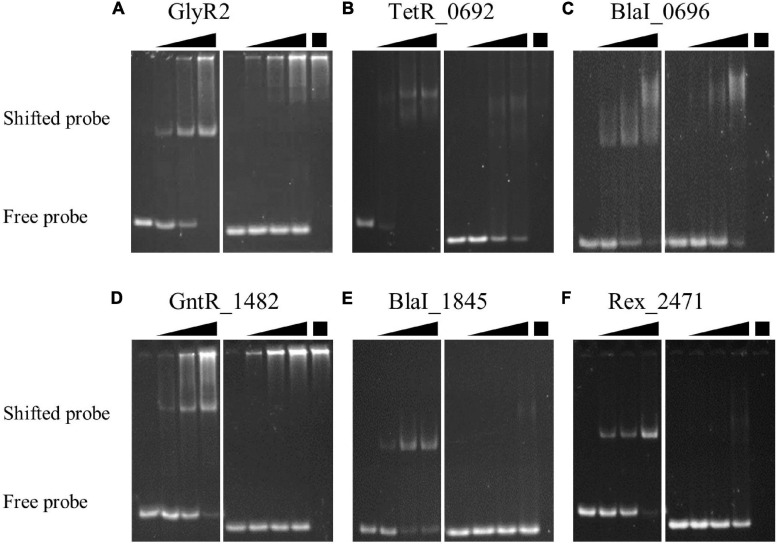
Sequence-specific binding of TFs to DNA. Single-step purified TFs were tested for binding to double-stranded DNA probes with or without the DAP-seq derived motif using EMSAs. Six TFs were of sufficient purity and quantity to demonstrate sequence specific binding: **(A)** GlyR2 (Clo1313_0089); **(B)** TetR _0692; **(C)** BlaI _0696; **(D)** GntR _1482; **(E)** BlaI _1845; and **(F)** Rex _2471. Free probes migrate further into the gel than probes in a TF-DNA complex. Gels for each TF were split into two subpanels to show binding to DNA probes with our identified motif (left) and to probes without the motif (right) of the respective TF. In each subpanel the first lane contains probe in the absence of protein. The subsequent three lanes include TF proteins in increasing concentrations represented by the black triangles above each subpanel. The final lane in the right subpanel repeats the highest concentration of protein (black box) but in the absence of probe DNA. This serves as a control to identify potential genomic DNA carried by some proteins during purification **(A,D)** to yield false positives. Probe sequences are available in Supplementary Table 6.

We used recombinant TFs and double-stranded DNA to test TF-DNA binding *in vitro*. Our EMSAs demonstrate sequence-dependent TF-DNA binding in favor of probes with their respective predicted motif for six of the 11 TFs (Figure 3). The remaining TFs showed low protein yield, purity and/or solubility after one-column purification and were therefore unable to be thoroughly tested in this study. GlyR2 (Clo1313_0089), TetR_0692, GntR_1482, BlaI_1845, and Rex_2471 shifted a substantial portion (>70%) of the positive probe without causing much of the negative probe to shift (<10%). The difference between positive and negative probes for BlaI _0696 was more modest (Figure 3C). These results confirm that the motifs derived from DAP-seq peaks indeed confer TF-DNA interactions.

In each EMSA, the concentration of probe was held constant, and the total protein concentration was varied to show titration of the protein-DNA interaction. Titrations of TF proteins against positive and negative DNA were performed on the same gel using the same TF concentrations, but they are split into separate sub-panels (Figure 3). The left sub-panel for each TF shows titration of a probe with the corresponding identified motif. The left lane has no added protein, and subsequent lanes increase in protein concentration. The right sub-panel shows a titration using the same concentrations of protein with a negative control probe. The last lane repeats the highest concentration of protein but without added probe. This lane provides a reference to distinguish *E. coli* genomic DNA (near the top of the lanes Figures 3A,D) from free or TF-bound probes (at the bottom and middle of the lanes, respectively, Figure 3). Test and negative control probe sequences for each EMSA are listed in Supplementary Table 6.

### The Role of DAP-Seq Peaks in Identifying TF Genome-Wide DNA Binding Sites

Having confirmed that the putative DNA binding motifs are selectively bound by TFs, we used these motifs to cross-validate the DAP-seq peaks. Each of these motifs encompasses an aligned set of similar sequences which can be represented quantitatively as a position-specific scoring matrix (PSSM). These PSSMs specify the likelihood of finding a particular nucleotide at each position in the motif sequence and can be used to identify TF binding sites. We set a requirement that all peaks in a regulon must have a TF binding site as identified by a significant match to the PSSM (*p* < 10^–4^). This requirement compensates for the cross-experimental variability of peaks in our DAP-seq experiments. Half of all peaks discovered in the high throughput screen contained the binding motif of their respective TF. This ratio improved to about three quarters in our repeat experiments. The use of five parallel replicates and lower throughput/multiplexing are factors that may contribute to this improvement. While we used motif-scanning to filter for relevant peaks, others have used RNA-seq to corroborate their findings from DAP-seq (Trouillon et al., 2021).

We also chose to only use peaks within non-coding regions for constructing TF regulons (Table 3). This selection was made to reflect the propensity of TFs for binding near or upstream of the promoters that they regulate and to simplify the analysis. We acknowledge that TFs may regulate gene expression by binding within the coding sequences of the genome. All DAP-seq peaks referred to hereafter are peaks that encompass at least one TF binding site and whose apex is positioned in a non-coding region. The complete regulons of individual TFs are discussed below and can be viewed in Supplementary File 1 and Supplementary Table 2.

**TABLE 3:** Summary of factors contributing to regulon reconstruction for each TF.

	DAP-seq peaks			Non-coding TF binding sites			
TF	Total peaks^a^	% with motifs^a^	in non-coding regions	By DAP-seq	By motif scanning	Total	Supporting evidence^b^
Xre_0026	604	45%	74	15	12	27	
GlyR2	686	45%	48	13	11	24	**E**, **F**, **H**
AraC_0222	552	96%	10	9	5	14	
TetR_0692	415	73%	25	17	4	21	**A**, **E**, **F**, **H**
BlaI_0696	51	73%	6	3	0	3	**A**, **E**, **F**
LexA_1449	850	44%	41	20	21	41	**A**, **F**, **H**
GntR_1482	423	62%	25	8	20	28	**E**
Fur_1691	587	53%	32	9	16	25	**F**
BlaI_1845	130	75%	4	1	0	1	**A**, **E**, **F**

*^*a*^DAP-seq peaks in both coding and non-coding regions.*

*^*b*^Additional evidence supporting the role of the TF include (***A***) evidence of autoregulation, (***E***) experimental validation of sequence specific DNA binding via EMSAs, (***F***) correlation between the predicted functions of the TF and its targets, and (***H***) homology between the TF and its motif with previously published counterparts.*

Different threshold parameters were required to determine putative TF-binding sites depending on the motif being used and the set of sequences being scanned. For most TFs, we used a threshold of *p* < 10^–4^ to identify binding sites in peak sequences and a threshold of *p* < 10^–5^ to identify binding sites in upstream gene regulatory sequences. However, these conditions were too strict for the BlaI_1845 motif. Two sequences, ATAGTCC and ATAGTCT, in nearly equal amounts comprise the BlaI _1845 motif, but a threshold of *p* < 10^–4^ recognizes only one of them. We therefore adjusted the cutoff for this PSSM to *p* < 2 × 10^–4^ to ensure that both sequences can be matched. Likewise, because the GlyR2 PSSM did not satisfactorily describe and identify known binding targets, we reconstructed the GlyR2 regulon using exact instances of GTAAnnTTAC or instances with up to one mismatch if they were in a peak in a non-coding region of the genome. The final predicted regulons of each TF and supporting evidence are given in Supplementary Table 2.

### Bioinformatic Inference of TF Functions

We also took comparative genomic approaches to evaluate the conservation of binding sites using footprint-scan from RSAT (Nguyen et al., 2018) or functional themes among target genes using GOMo (Buske et al., 2010). These methods evaluate the conservation of TFs, target genes, and DNA binding sites among several genomes. Five of the 11 TF-motif pairs used for our footprint-scan analysis identified conserved elements. The LexA _1449 and Rex2471 TFs had the largest and most widely conserved set of potential target genes (10 and 14 genes, respectively). Two potential target genes were conserved for BlaI_0696and Fur_1691, respectively. The only conserved regulatory interaction for TetR0692 was self-regulatory. In fact, self-regulatory mechanisms, where the TF targets its own gene, were conserved for four of these five genes (LexA_1449, Rex_2471, BlaI_0696, TetR_0692, with Fur_1691 being the exception). The conserved target genes for each TF are addressed individually in the next section and listed in Supplementary Table 2. The lack of results for the other TFs may be a result of having species-specific roles of the species selected for the comparison, novelty of the TF, or could be simply that the combination of parameters (Nguyen et al., 2018) fell outside of the detectable ranges.

Our searches for conservation of specific functions using GOMo were subject to the same limitations and had similar performance as the footprint-scan from RSAT. Conserved functions (*p*-adj < 0.05) were found among genes with high scores for the LexA_1449, Rex_2471, TetR_0692, and GntR_1482 motifs. A single gene ontology term for SOS response to DNA damage was found for LexA_1449. The Rex_2471 motif was upstream of genes enriched in ontology terms for redox activity, glycolysis, and transcription regulation. The other motifs each identified transposition and nucleic acid binding as conserved target functions. Transposition was additionally associated with the TetR_0692 motif. Because individual binding sites are not identified by GOMo, we did not attempt to infer individual target genes from this search. However, these functional themes generally agree with our phylogenetic footprinting experiments and the predicted functions of these TFs.

### TF Regulatory Networks

We used a combination of experimental and bioinformatic methods (Figure 1) to gain insights into the regulons of putative TFs in *C. thermocellum*. While tens of peaks—up to nearly a thousand peaks—were found for each TF in the genome-wide assay, repeat experiments showed high variability in the count, location, and relative sequencing depth of these peaks (Table 2 and Supplementary Figure 1) but converged to the same binding sequence motifs. We thus overcame this variability by using TF DNA binding motifs (Figure 2) with *in vitro* validation (Figure 3) to evaluate the potential of the TF to bind to DAP-seq peaks and other non-coding sequences not captured by DAP-seq peaks in the genome. The iteration of experimental and bioinformatic approaches was especially important for GlyR2 (Clo1313_0089), which will be discussed first. We will then discuss the regulons of other TFs generally ordered by size from the small regulons for the BlaI TFs (BlaI_0696, BlaI_1845) and Xre_2225 to the large regulons of LexA_1449 and Rex_2471.

#### LacI-Type TF GlyR2 (Clo1313_0089) Regulates Several Mannanases and Cellulosomal Enzymes

The three LacI repressor-type TFs encoded in *C. thermocellum*, GlyR1, GlyR2, and GlyR3 (Clo1313_2023, Clo1313_0089, and Clo1313_0396, respectively), are of particular interest for their involvement in regulating central carbon metabolism. LacI TFs alter between DNA binding and non-binding conformations in response to the absence or presence of a specific ligand, usually a sugar molecule. All three *C. thermocellum* LacI proteins were tested in our large-scale screen but no DAP-seq peaks were detected under this condition. We were, however, able to identify peaks for GlyR2 from a targeted DAP-seq experiment.

In a previous report, 17 genes were upregulated (potentially de-repressed) in a *glyR2* deletion strain (Wilson et al., 2017). Direct binding was demonstrated between GlyR2 and a PCR fragment amplified form the 58th to the 207th nucleotide upstream of the most upregulated gene, *clo1313_1398* (Wilson et al., 2017). The gene *clo1313_1398* encodes Man5A, a cellulosome protein with a mannanase enzyme domain. Although our DAP-seq experiments did not identify a peak upstream of the *man5A* gene, the most abundant peak selected by GlyR2 was upstream of *clo1313_0399*, another cellulosomal mannanase (*man26A*) that was upregulated in the *glyR2* mutant (Wilson et al., 2017). This evidence of protein-DNA interaction suggests that the observed differential expression of Man26A is also likely due to direct regulatory interactions.

We consider the absence of a peak upstream of *man5A* (*clo1313_1398*), where binding and regulation had been previously observed, to be a false negative DAP-seq result. However, DNA binding motifs upstream of *man5A* and other potential false negatives were recaptured by the bioinformatic motif searches. The best match to the GlyR2 PSSM (Figure 2B) in the known binding region was excluded by our threshold of *p* < 10^–4^ but included the sequence GTAATATTAC. This sequence contains two inverted repeats of GTAA found in the GlyR2 motifs (Figure 2B and Supplementary Figure 2B) and in the double-stranded DNA probe bound by GlyR2 in EMSAs (Figure 3A and Supplementary Table 6). Because we could not identify a suitable threshold that would select for the GTAAnnTTAC sequence observed in these known binding sites, we resorted to simple sequence searches to identify potential binding sites for GlyR2. This sequence is upstream of 16 operons (23 genes) encoding six enzyme components of the cellulosome. Three of these were upregulated in the *glyr2* mutant, which are *man5A* and *man26A* mentioned earlier and a cellulase *celE* (*clo1313_1425*). The other three cellulosome components with GlyR2 binding sites are glycoside hydrolases family 5 (*clo1313_0413*), a xylanase (*clo1313_2530*), and a lipolytic enzyme (*clo1313_1783*).

These findings are consistent with the functional role of GlyR2 as a mannobiose-responsive transcription repressor (Wilson et al., 2017). We have shown that GlyR2 binds to a sequence motif upstream of genes encoding cellulosome enzymes and other genes. Our findings suggest that three of the genes that were differentially expressed in a GlyR2 mutant strain are directly regulated by GlyR2. The absence of a GlyR2 binding site upstream of the remaining genes may indicate that their differential expression was indirectly related. The presence of the GlyR2 upstream of non-differentially expressed genes also presents a likelihood that these genes may be repressed by GlyR2 but require different conditions to activate transcription. These findings refine the current model of transcription regulation by GlyR2 in *C. thermocellum*.

In the case of the GlyR2 regulon, our bioinformatics results played the role of expanding the regulon to include genes that were missing from the DAP-seq data. For the next three regulons that we discuss, our bioinformatics results play a nearly opposite role. The sequence scanning results for the two BlaI TFs (BlaI_0696, BlaI_1845) and Xre_2225 provided evidence to explain why so few DAP-seq peaks were within non-coding sequences. In one case these results also provide justification for eliminating a gene identified by DAP-seq from the regulon.

#### Small, Well-Defined Regulons of BlaI _0696 and BlaI _1845

BlaI_0696 and BlaI_1845 belong to the penicillinase/beta-lactamase repressor (BlaI) family of TFs. BlaI TFs often regulate their own operon and operons containing beta-lactam resistance genes by binding as a dimeric complex to inverted repeat DNA sequences (Zhang et al., 2001; Golemi-Kotra et al., 2003; Sala et al., 2009). Only two and three peaks for the respective BlaI TFs were in non-coding regions. A peak indicative of self-regulation was found for each of these TFs. The representative motif for BlaI_0696 contains inverted repeats of GTCTAT five nucleotides apart (Figure 2E). The BlaI_1845 motif contains a single repeat of a similar sequence GACTAT (Figure 2I). We confirmed that these TFs bind to probes with single repeats of their respective motifs using EMSAs (Figures 3C,E and Supplementary Table 6).

**FIGURE 4:**
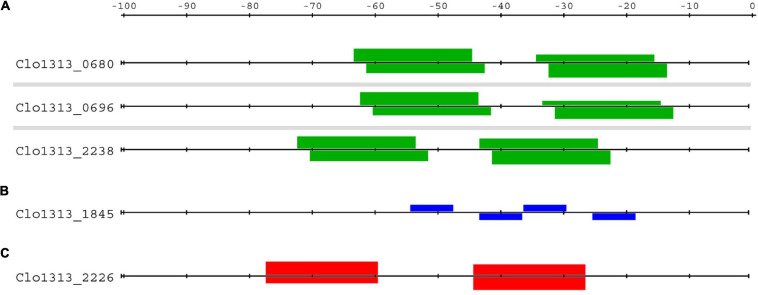
Distribution of BlaI and antitoxin binding sites upstream of target genes. Highly ordered sets of binding sites were found upstream of the putative gene targets of **(A)** BlaI BlaI_0696, **(B)** BlaI_1845 and **(C)** Xre_2225. Each horizontal line represents the sequence upstream of the locus tag on its left. Boxes above or below represent binding sites of the respective TF on the coding or template strand, respectively. The bindings sites for BlaI_0696 (green boxes, **A**) and Xre_2225 (red boxes, **C**) contain inverted repeats (Figure 2), whereas the BlaI_1845 binding site (blue boxes, **B**) contain only a single repeat. The height of the box references the *p*-value of the binding site, the taller the box the lower the *p*-value.

Although only DNA probes with single binding sites were tested in EMSAs with these BlaI TFs, our bioinformatic screening for genomic binding sites provides ample evidence that BlaI_0696 and BlaI_1845 bind to DNA as dimers *in vivo*. In fact, this evidence also suggests that these BlaI TFs target genes with highly ordered binding sites for four protomers (Figure 4). Only three of the BlaI_0696 DAP-seq peaks were in upstream non-coding regions (upstream of *clo1313_0680*, *clo1313_0696*, and *clo1313*_2238). These peaks each contained two matches to the BlaI_0696 representative motif (green boxes, Figure 4A). Because each motif represents binding of two TFs to inverted repeats (Figure 2E), these targets can potentially be bound by up to four TFs simultaneously. Two DAP-seq peaks in non-coding regions were found for BlaI_1845, but only the peak upstream of the BlaI_1845-encoding operon had matches to the BlaI_1845 motif (Figure 2I). Four instances of the BlaI_1845 motif are upstream of its own operon (blue boxes, Figure 4B), structured as pairs of inverted repeats similar to the pattern observed for BlaI_0696 targets. This pattern of four TFs binding to two pairs of inverted repeats appears to be a strict requirement for regulation by these BlaI TFs. No other genes have more than a single binding site for either TF. We therefore conclude that the BlaI_0696 and BlaI_1845 regulons contain only these three and one operons, respectively, represented in Figure 4.

The functions of the genes in the BlaI_1845 and BlaI_0696 regulons are consistent with a response to beta-lactams. Beta-lactam antibiotics achieve bactericidal activity by irreversibly inhibiting peptidoglycan synthesis enzymes. The resulting inability to restructure the cell wall is fatal for the bacteria. Cells avoid beta-lactam toxicity by deploying either beta-lactamase enzymes or alternative cell wall synthesis enzymes. Beta-lactams are sensed by BlaR1 proteins. In the presence of beta-lactams, BlaR1 proteolytically cleaves BlaI, thus relieving inhibition of the BlaI regulon (Zhang et al., 2001; Golemi-Kotra et al., 2003). The single operon forming the BlaI_1845 regulon contains genes encoding BlaI_1845, BlaR1 and two hypothetical proteins, one of which is a putative homologue of *Nitrosococcus oceani* peptidoglycan synthesis gene Noc_2864. This operon contains genes with sensory (*blaR1*), transduction (*blaR1* and *blaI*), and response functions (peptidoglycan synthesis) apparently sufficient to limit the effects of beta-lactams. The *clo1313_0696* operon consists of genes encoding BlaI_0696, two BlaR1s, and a hypothetical protein (Clo1313_2238). Since all other genes in this regulon are involved in sensory and signal transduction functions, we suspect that *clo1313*_2238 encodes the antibiotic response mechanism.

#### Autoregulatory Role of Xre_2225 in a Putataive Type II Toxin-Antoxin Module

Like BlaI_1845, only a single peak for Xre_2225 was in a non-coding region. This peak is upstream of the *clo1313_2226*-*clo1313_2225* operon, encoding a Type II toxin-antitoxin pair, respectively (Xie et al., 2018; Akarsu et al., 2019). Free toxins cause growth arrest or cell death by targeting genome replication or protein translation. Antitoxins bind to and neutralize the effects of toxins (Page and Peti, 2016). Antitoxins, whether free or in complex with the toxin, also act as transcriptional repressors of the TA genes. It is, therefore, fitting that the sole regulatory true-positive peak for antitoxin Xre_2225 should be upstream of its own operon.

Only six matches to this PSSM (*p* < 10^–5^, Figure 2J) are in regulatory regions in the genome. Two of these matches have much higher scores than the rest (equivalent to *p* ∼ 10^–8^ vs. *p* ∼ 10^–5^), and both of these high scoring sites lie upstream of *clo1313_2226* (Figure 4C). These two matches have better scores because they adhere to the consensus sequence of TACATCnnnnnnGATGTA, while all others have at least one mismatch in each of the TACATC half sites. The unique dual binding sites upstream of *clo1313_2226* may be a trait selected for by evolution. The presence of competing binding sites elsewhere in the genome would compete for the repressor resulting in higher transcription from the toxin-antitoxin operon. Because antitoxins are generally more labile than their toxin partner, this would result in a higher and perhaps lethal equilibrium concentration of toxin. We reason that Xre _2225 regulates only its own operon by binding as a dimer to inverted repeats of TACATC.

#### Predicted Regulon of TetR_0692

TetR_0692 is a member of the TetR family of transcriptional regulators, which was named after a tetracycline-sensitive repressor. The model TetR represses transcription of its own gene and a divergently transcribed tetracycline efflux pump (Ramos et al., 2005). It was shown that transcriptional repression is achieved by dimeric TetR binding to a palindromic sequence overlapping the promoters of the two divergent operons (Ramos et al., 2005; Bertram and Hillen, 2008). TetR family TFs are best known for regulating efflux pumps, but they have been shown to regulate a wide variety biological functions in response to various classes of ligands (Cuthbertson and Nodwell, 2013).

A DAP-seq peak was upstream of TetR_0692 in both experiments, with apices at 59 and 31 bp upstream of the coding sequence. These apices mark two of the top three scoring positions in all regulatory regions in the genome. The -31 position marks a perfect palindrome TGACCACGTGGTCA, the only one in the entire genome. EMSAs confirmed that TetR_0692 binds to a 36 bp oligo containing this palindrome (Figures 2D, 3B and Supplementary Table 6). These results provide strong evidence that TetR_0692 operates an autoinhibitory transcriptional feedback loop similar to the model TetR regulatory system from Enterobacteriaceae (Tovar et al., 1988). It is unclear whether binding to these sites will co-regulate transcription of the divergently transcribed gene approximately 200 bp away, which encodes a protein with putative carbohydrate-binding, dockerin, and lipolytic domains.

In the cases of BlaI_0696, BlaI_1845 and Xre_2225 discussed earlier, the pattern of regularly spaced binding sites found upstream of the TF-encoding gene served as a criterion for including other genes in the regulatory network. Although the TetR binding site upstream of its own gene is unique, we do not conclude that the TetR regulatory network is limited to genes downstream of this binding site. The putative regulatory binding sites that we identified using BlaI_0696, BlaI_1845, and Xre_2225 motifs spontaneously fell into one of two very different categories. They occurred either as individual, low-scoring events outside of peak sequences or as regularly spaced clusters of high-scoring events inside peak sequences. The putative binding sites for TetR_0692, and the remaining TFs in this report, are not divided into such a binary distribution. The binding sites of the remaining TFs in this report generally show a continuum of scores even among those sites inside DAP-seq peaks. The disparity between binary and continuous distributions of scores arises, bioinformatically speaking, from the stringency of the DAP-seq derived motifs. The motifs found for the BlaI TFs among all combined peak sequences allowed for very little deviation from the consensus sequence as indicated by the near maximal height of all letters in the motifs (Supplementary Figure 2A). Most motifs for other TFs have several positions that can be occupied by different nucleotides (Figure 2 and Supplementary Figure 2A). From a biological perspective, it may be advantageous to limit the BlaI and antitoxin TFs to tightly repress a few genes that can compromise a healthy cell, whereas there is little resistance to the acquisition of target genes for the other TFs (Sorrells and Johnson, 2015).

Consistent with the established knowledge of TetRs, TetR_0692 binding sites (Figure 2D) and DAP-seq peaks are upstream of two putative efflux proteins (*clo1313_1168* and *clo1313_2874*). Other predicted binding sites and DAP-seq peaks are upstream of genes with roles in genome methylation and maintenance, genes encoding components of the ribosome, and a large operon encoding cell wall synthesis enzymes involved in vancomycin resistance.

#### Fur_1691 Targets a Zinc Transporter

As a member of the ferric uptake regulator protein family, Fur_1691 is anticipated to regulate uptake and homeostasis of iron, zinc, or magnesium ions. Ten DAP-seq peaks were identified in both DAP-seq experiments. The highest fold change peak is upstream of a zinc-specific ABC transporter operon (*clo1313_1690*-*1688*). Two binding sites occur at the apices of these peaks forming a pseudo-palindromic sequence TGCAAATcATTTGCA (Figure 2H). Similarly high scoring binding sites are conserved upstream of zinc transporters in *Clostridium clariflavum*, *Clostridium stercorarium*, and *Caldicellulosiruptor bescii*, indicating a conserved role of Fur _1691 and its orthologs as zinc uptake regulators. A model zinc uptake regulator (Zur) from *E. coli* binds to and represses transcription of a zinc-specific ABC transporter and other genes in the zinc-bound state (Graham et al., 2009; Gilston et al., 2014). Under zinc-deficient conditions zinc-free Zur release DNA derepressing these genes. In some organisms, Zur also activates zinc exporters when the zinc ion concentration is too high (Huang et al., 2008; Choi et al., 2017b). The conserved presence of a Fur _1691 binding site upstream of the zinc-specific ABC transporter are in good agreement with the role of the model *zur*. Although we did not test it directly, we expect Fur _1691 to also be responsive to zinc.

Other putative gene targets worth noting include a mechanosensitive ion channel (*clo1313_0094*) and the Type II toxin-antitoxin operon mentioned earlier (*clo1313_2226*-*clo1313_2225*). Coupling transcription of a mechanosensitive ion channel to zinc transport may help maintain osmotic balance. The control of a Type II TA system by a zinc uptake regulator could provide a mechanism for cells to enter a non-growth state until there is a change in their environment. However, an additional layer of transcriptional repression control on the TA system may provide a more compelling benefit than zinc deficiency-coordinated expression.

#### Enigmatic Regulons of GntR_1482, Xre_0026, and AraC_0222

The regulons of GntR_1482, Xre_0026, and AraC_0222 are not as robustly supported as the other regulons reconstructed in this report. The motifs and bioinformatic searches for the other TFs have led to results that, in hindsight, are at least somewhat anticipated: a ferric-uptake regulator targeting metal ion homeostasis; a version of the GlyR2 motif being present in a known target; transcriptional autoregulation by BlaI TFs and a putative antitoxin (Xre_2225). The results for the three TFs discussed in this section do not seem to converge and coalesce to the same extent.

The GntR_1482 and Xre_0026 motifs both feature elements of dyad symmetry commonly found in TF binding sites (Figures 2A,G, respectively). The AraC_0222 motif (Figure 2C) does not have such features, but it is notable for how highly enriched it is among the underlying DAP-seq peaks and for how consistently the peaks were identified across experiments (Supplementary Figure 2A). We were able to demonstrate sequence-specific binding by GntR_1482 (Figure 3D), confirming the association between the TF and the derived motif. This motif is enriched upstream of genes encoding proteins with DNA-binding activity including three TF-encoding target genes (a putative fatty acid synthesis regulator *clo1313_1279*, *clo1313*_0692, and a two-component transcriptional regulator *clo1313_2868*) and a few genes involved in DNA replication (replicative DNA helicase *clo1313_2929*, a transposase *clo1313_2369* and a phage DNA polymerase-related protein encoded by *clo1313_0695*). The GntR_1482 regulon also contains *clo1313_1372*, a peptidase M24, and *clo1313_1170*, a encoding hypothetical protein. The peptidase gene was the only gene to be identified as a GntR_1482 target in both DAP-seq experiments, and both of these genes have two putative binding sites in their upstream regions. The rest of the regulon is comprised of genes of various predicted functions and can be found in Supplementary Table 2.

We were unable to confirm binding by Xre_0026 or AraC_0222 to their respective motifs. There was also no evidence of autoregulation or any other discernable bioinformatic evidence associating the TFs to their respective motifs. AraC_0222 does not have a discernable DNA binding domain, but rather it appears to be the N-terminal fragment of an AraC protein encoded by a fusion of *clo1313_0222* and *clo1313_0223* gene products via a programmed translational frame shift. However, the AraC_0222 DAP-seq results were the most consistent of all samples (Supplementary Figure 1), and improvements in protein purification could also improve the performance of Xre_0026 in EMSAs. We therefore briefly present these regulons, acknowledging the associated caveats.

The putative AraC_0222 regulon includes three DAP-seq peaks targeting *clo1313_2506*, *clo1313_2515*, and *clo1313_2523*. These genes encode nearly identical hypothetical proteins and are parts of repeating genomic neighborhoods that encode copper amine oxidases, putative zinc-binding lipoproteins, DNA helicases and transposases. We are unsure of the role that these targets may have, but they are noteworthy for their reproducibility and higher fold change relative to other DAP-seq peaks. Among the other potential target genes inferred by DAP-seq or bioinformatic experiments are a DegS/DegU-like two-component signal transduction system, histidyl-, aspartyl-, and glutamyl-tRNA synthetases. The DegS/DegU two-component system in *Bacillus subtilis* regulates systems involved in the transition from exponential phase to stationary phase (Mader et al., 2002) which would coincide with a decrease in protein synthesis. From these target genes it seems that the TF associated with this motif may provide input to life-cycle progression processes and warrants further investigations.

The predicted Xre_0026 regulon includes several genes related to sporulation (sporulation integral membrane protein *clo1313_1202*, *sleB clo1313_0046*, *ftsK*/*spoIIIE clo1313_1118*, and germination protein YpeB c*lo1313_0047*). The correlation between the Xre_0026 motif and sporulation genes is not quantitative, and these genes are a small portion of the predicted target genes. The full list is available in Supplementary Table 2.

#### DNA Damage and SOS Response Regulated by LexA_1449

DAP-seq peaks (with detected binding sites) for LexA_1449 were found in upstream non-coding regions in both experiments. The DNA binding sequence motif derived from all DAP-seq peaks matches the *Bacillus subtilis* LexA motif including the dyad symmetry (Figure 2F; Au et al., 2005). We were unable to confirm binding of LexA_1449 to its motif because of heavy protein precipitation under these conditions for protein purification. However, the high similarity of LexA_1449) and its motif to published protein-DNA motif pair confirms the DAP-seq results.

LexA proteins, includingLexA_1449, have a peptidase domain that catalyzes autolysis in the presence of RecA and single-stranded DNA leading to de-repression of DNA damage repair genes (Butala et al., 2009). The LexA_1449 regulon that we reconstructed is consistent with this role and includes RecA (Clo1313_1163), DNA repair proteins (Clo1313_0599 and Clo1313_2742), subunits A and B of an excinuclease (Clo1313_1916 and Clo1313_1918) and DNA gyrase B (Clo1313-1922). Three binding sites are also upstream of the LexA-encoding gene (*clo1313_1449*), as was also the case for *B. subtilis* LexA (Au et al., 2005). These sites enable a negative feedback loop which is important for a rapid and evolutionarily resilient response (Marciano et al., 2014; Kozuch et al., 2020).

Except for the DNA repair protein encoded by *clo1313_0599*, all these genes were identified as conserved regulatory targets in our footprint-scan experiments. Four other genes were also identified with conserved binding sites (Supplementary Table 2), the most noteworthy being *clo1313_1450* which encodes a protein with a domain structure similar to YneA in *B. subtilis*. YneA contains a peptidoglycan-binding lysin domain and appears to inhibit cell division as part of the SOS response to DNA damage (Mo and Burkholder, 2010). The genes, encoding Clo1313_1450 and LexA_1449, are divergently transcribed and share upstream non-coding regions similar to *yneA* and *lexA* in *B. subtilis*. This may be a common theme as it was also present among the organisms where the LexA_1449 binding site was found upstream of orthologs of Clo1313_1450. In all, these data describe the role of LexA_1449 in regulating the SOS response to DNA damage.

#### Role of Redox Sensitive TF Rex _2471 in Regulating Central Carbon Metabolism and Redox Homeostasis

The Rex regulon appears to be highly variable, but generally pertains to pyruvate fermentation, NAD-dependent energy metabolism (Ravcheev et al., 2012). In Clostridia, Rex regulons include primarily ethanol/butanol and butyrate production genes (Zhang et al., 2014) downstream of a Clostridiaceae-specific consensus sequence of TTGTTAANNNNTTAACAA (Ravcheev et al., 2012). The DAP-seq derived Rex_2471 motif matches this consensus (Figure 2K) and was validated by EMSA (Figure 3F). This motif is significantly associated with genes involved in glycolysis, redox activity, and transcription factor activity (GOMo, *q* < 10^–3^). Fourteen target genes were also predicted to be part of a conserved regulon using footprint-scan from RSAT (Table 4). Nine of these genes are orthologs of genes in the *C. bescii* Rex operon (Table 4), including five genes in a hydrogenase-encoding operon (*clo1313_1885*- *clo1313_1881*), a putative hydrogenase system transcriptional regulator (*clo1313_1572*) and Rex_2471 itself (Sander et al., 2019). The hydrogenase transcriptional regulator was not identified in DAP-seq but was found through PSSM scanning (*p* < 10^–5^) and footprint-scan, demonstrating the complementarity between these two approaches. The fact that an interaction between Rex and this hydrogenase regulator was previously observed in the literature (Sander et al., 2019) confirms that it is part of the Rex_2471 regulon.

**TABLE 4:** Highlights of the Rex_2471 regulon.

Target	Predicted function	*C. bescii* ortholog(s)^a^	DAP-seq peak	Predicted binding sites
Clo1313_0020-23	Pyruvate ferredoxin oxidoreductase	Athe_0874-77	+	1
Clo1313_0530	Energy-coupling factor transport protein		+	1^b^
Clo1313_0586	2-hydroxyacid dehydrogenase	Athe_2125	+	2^b^
Clo1313_1571	Hydrogenase maturation enzyme, hydG	Athe_0169	+	1^b^
Clo1313_1572	Putative iron-only hydrogenase system transcriptional regulator	Athe_0168	−	1^b^
Clo1313_1686	Acetyl-CoA synthetase		−	1
Clo1313_1875	Aldolase		+	2^b,c^
Clo1313_1876	Phosphofructokinase		+	2^b,c^
Clo1313_1878	Lactate/malate dehydrogenase		−	1
Clo1313_1879	Malic enzyme		−	1
Clo1313_1885-81	Iron-only hydrogenase	Athe_1295-99	+	1^b^
Clo1313_1944	Isocitrate dehydrogenase		+	1^b^
Clo1313_2015	Glucose-6-phosphate isomerase		+	1
Clo1313_2090	Enolase		+	1
Clo1313_2093	Triosphosphate isomerase		−	1
Clo1313_2094	Bifunctional phosphoglycerate kinase/trisophosphate isomerase		−	1
Clo1313_2095	Glyceraldehyde-3-phosphate dehydrogenase		+	1^b^
Rex_2471	Redox-responsive transcription regulator Rex	Athe_0654	+	1^b^

*^*a*^Only orthologs that are part of the Caldicellulosiruptor bescii Rex regulon are listed. Orthology is determined using a bidirectional best BLAST hits criterion.*

*^*b*^These Rex_2471 binding sites were also enriched upstream of orthologous genes.*

*^*c*^The aldolase and phosphofructokinase encoded by Clo1313_1875 and clo1313_1876 are divergently transcribed and share an upstream region that contains two binding sites. It is unclear whether each site regulates a different gene or if both sites act on one or both genes.*

The first Rex TF was characterized for its response to oxygen deprivation in *Streptomyces coelicolor*, where it represses transcription until the NADH/NAD^+^ ratio rises above a given threshold (Brekasis and Paget, 2003). Rex proteins in clostridia are similarly responsive to the redox balance of the NAD pool (Wietzke and Bahl, 2012; Zhang et al., 2014; Sander et al., 2019). Rex_2471 is one of two genes annotated as redox-sensitive Rex-type transcriptional regulators in *C. thermocellum*. Rex_2471 is more similar (60 and 59% amino acid identity) to the characterized Rex proteins from *Clostridium acetobutylicum* (Wietzke and Bahl, 2012; Zhang et al., 2014) and *Caldicellulosiruptor bescii* (Sander et al., 2019) than is Rex_1799 (36 and 34%). However, Rex Cthe_0422 from *C. thermocellum* ATCC 27405 (homolog of Rex_1799) was downregulated under furfural-induced stress while Cthe_1798 (homolog of Rex_2471) had no detectable change (Wilson et al., 2013b). This pattern, where the Rex_2471 homolog was unchanged while the Rex_1799 homolog was differentially expressed, was also observed in a poplar hydrolysate-tolerant strain (Linville et al., 2013, 2014). This may suggest that Rex_1799 is more sensitive to environmentally induced stress than Rex_2471.

**FIGURE 5:**
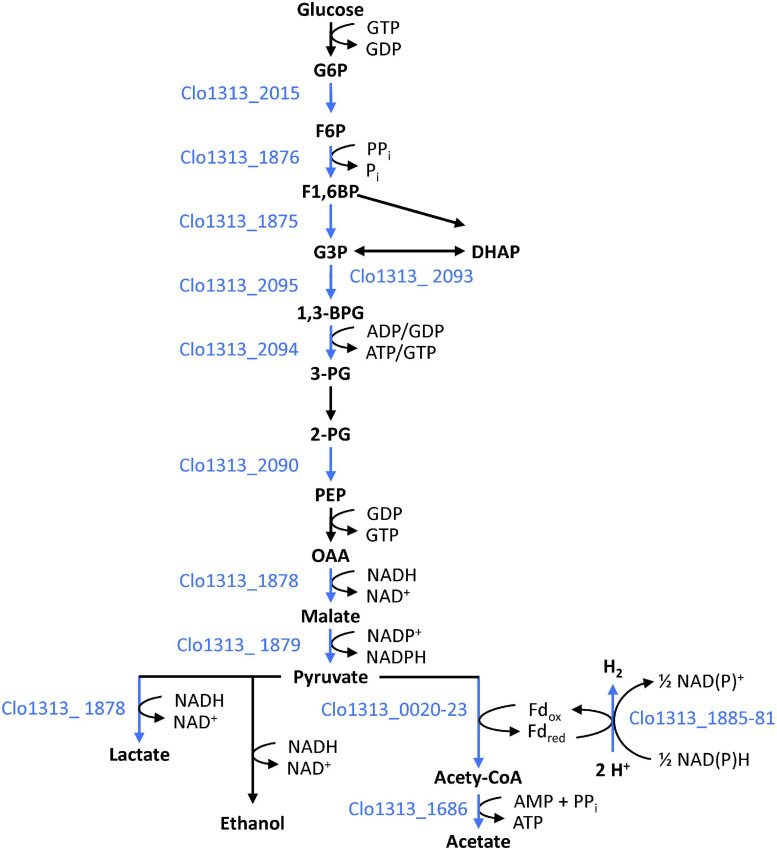
Role of Rex_2471 in regulating glycolysis. The Rex_2471 binding site appears upstream of genes encoding enzymes that account for most steps in glycolysis. Locus tags encoding enzymes downstream of Rex binding sites are shown to the left or below arrows depicting the step they putatively catalyze. Blue text and blue arrows indicate steps putatively regulated by Rex_2471. See also Table 4 for more details. Cofactors to the right of arrows indicate the general specificity observed in Zhou et al. (2013).

In addition to the hydrogenase system, Rex_2471 appears to regulate glycolysis and fermentation. DAP-seq peaks and/or high-scoring binding sites are upstream of genes encoding enzymes that catalyze most of the steps in glycolysis (Figure 5). Three glycolysis genes (aldolase, phosphofructokinase and glyceraldehyde-3-phosphate dehydrogenase, Table 4) had conserved binding sites upstream of orthologous genes in other organisms, though conservation of these binding sites was not so wide-spread as the hydrogenase system mentioned above. Rex_2471 binding sites were also upstream of genes whose products catalyze reactions branching from or downstream of glycolysis. Some of the proteins encoded by these genes such as a PFOR complex (Clo1313_0020-Clo1313_0023), 3-phosphoglycerate dehydrogenase (Clo1313_0586) and isocitrate dehydrogenase (Clo1313_1944) would produce reducing equivalents (NADH or typically NADPH for the case of isocitrate dehydrogenase) during consumption of cellobiose, while others would consume reducing equivalents: lactate dehydrogenase (Clo1313_1878), two short-chain dehydrogenases (Clo1313_0720 and Clo1313_0815), and hydrogenases (Clo1313_1885-Clo1313_1881 and Clo1313_0554).

The recycling of reduced NADH to oxidized NAD^+^ is necessary for continued glycolysis and other cellular functions. The canonical role of Rex is to limit the expression of NADH-recycling enzymes, such as hydrogenases and alcohol dehydrogenases, according to the redox balance of the NAD pool. When the NADH/NAD^+^ ratio rises, Rex releases DNA to derepress the transcription of NADH-recycling enzymes until the redox balance is restored. Under this paradigm, it is fitting that Rex_2471 targets enzymes that utilize NADH such as those listed in the previous paragraph. It also seems counter-intuitive for Rex_2471 to regulate NADH-producing enzymes, such as those involved in glycolysis, in the same way. Rex binding sites have previously been observed upstream of genes encoding glycolytic enzymes (Ravcheev et al., 2012), but to our knowledge the role of Rex in regulating glycolysis has not yet been studied experimentally. The possibility that Rex could act as a transcriptional activator has been suggested, but not yet demonstrated (Bitoun et al., 2011, 2012; Bitoun and Wen, 2016). There is much that remains to be known about the role of Rex transcription factors in *C. thermocellum*. The putative regulon described herein (Table 4 and Supplementary Table 2) provides a foundation for future studies and engineering involving Rex TFs in *C. thermocellum*.

### Structural Features of the Transcriptional Regulatory Networks

**FIGURE 6:**
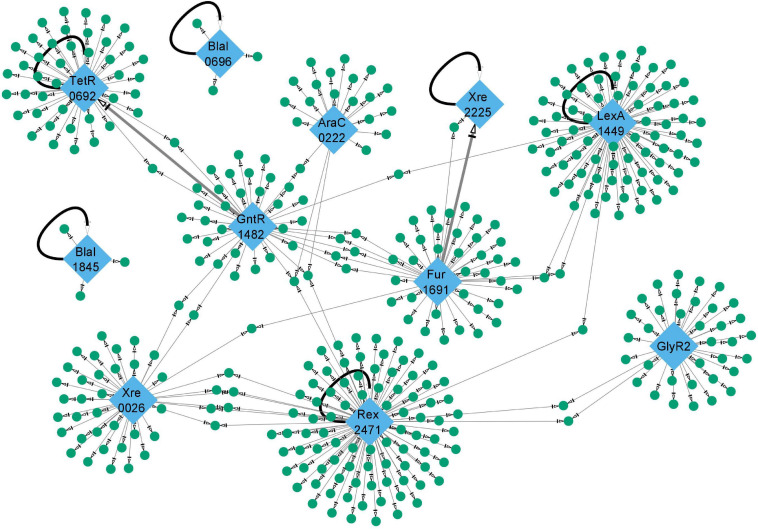
Size and interconnection of gene regulatory networks in this work. The gene regulatory networks described in the main text are shown. Transcription factors are shown as blue diamonds and target genes as green circles. The two BlaI TFs (BlaI_0696 and BlaI_1845) and Xre_2225 regulate small subnetworks while Rex_2471 and LexA_1449 regulate a much more expansive set of genes. These five TFs mentioned so far, along with TetR_0692, appear to repress transcription of their own gene as depicted by bold black curved arrows. Two bold gray arrows indicate potential hierarchical regulatory interactions between TFs characterized in this study (GntR_1482 acting on TetR_0692, and Fur_1691 acting on Xre_2225). The complete network can be viewed in Supplementary File 1 and Supplementary Table 2.

The 11 individual TF regulons constitute subnetworks in a global network that connects 437 distinct genes in *C. thermocellum* (Figure 6). This network includes a highly specialized TF (Type II toxin-antitoxin system) regulating only two genes including itself, and TFs regulating large subnetworks such as Rex_2471, and LexA_1449 which control 114, and 90 genes, respectively (Supplementary Table 2). Based on the gene networks reconstructed in this study, the global network displays hierarchies of transcriptional regulations as well as multi-transcription factor regulation of gene targets. As described, Rex_2471 appears to be a global regulator higher in the regulatory hierarchy controlling 7 TFs including itself and iron hydrogenase transcription regulator (Clo1313_1572). These TFs are from various families with broad biological roles. It remains to be seen how Rex_2471 exerts targeted effect, and if and how those Rex_2471-regulated TFs contribute to cellular redox homeostasis. In addition, two of the TFs, TetR_0692 and the regulator of Type II toxin-antitoxin system (Xre_2225), are both regulators as well as regulatory targets identified for GntR_1482 and Fur_1691, respectively. Interestingly, a GntR_1482 binding site overlaps with one of the autoregulatory TetR_0692 binding sites, potentially destabilizing the control of TetR_0692 over its own gene. Likewise, a Fur_1691 binding site is in a position that would compete with Xre_2225 upstream of the toxin-antitoxin operon. It is unclear whether this competition would trigger transcription or if the Fur_1691 repressor acts as a backup repressor to limit toxin-antitoxin transcription. There were also around 30 genes that appeared to be targeted by two or more TFs (Figure 6 and Supplementary File 1).

Also observed in this global gene network are six TFs which appear to negatively regulate the transcription of their own gene: TetR_0692, BlaIs (BlaI_0696 and BlaI_1845), LexA _1449, Xre_2225, and Rex _2471. Negative self-regulation minimizes the basal expression of a TF and reduces noise in transcription (Becskei and Serrano, 2000). It seems that a smaller population of TF is readily saturated with the incoming signal and is more capable of a rapid response (Kozuch et al., 2020). This helps cells to conserve resources and deploy them rapidly and precisely at the time of need.

## Conclusion

This work is a substantial addition to the body of knowledge pertaining to transcription factors and gene regulatory networks in *Clostridium thermocellum*. To our knowledge, this is also the first report of large-scale, DAP-seq screening of bacterial TFs. This systems biology approach expedites the discovery of multiple TFs’ DNA binding sequence motifs and subsequently their regulatory targets, thereby revealing regulatory information not previously known. Our bioinformatic searches served to both remove false positive peaks and identify novel and anticipated members of the regulon that were missing from the DAP-seq data. This highlights the complementarity of the DAP-seq and bioinformatic analyses. Coupling with traditional EMSA to validate DAP-seq and bioinformatic findings, we established a framework to transform large sets of data from DAP-seq to physiologically relevant information. Overall, the TFs characterized herein and their respective regulons provide a valuable foundation for future scientific and engineering endeavors.

## Data Availability Statement

The datasets presented in this study can be found in online repositories. DAP-seq raw sequencing reads that support the findings of this study have been deposited in the Sequence Read Archive with the BioProject ID PRJNA723898. An interactive Cytoscape file containing the complete gene regulatory networks and supporting evidence summarized in this work will be made available through the NREL Data Catalog, accessible at https://data.nrel.gov/submissions/161. The NREL Data Catalog is the electronic catalog for data generated by federally funded research at the National Renewable Energy Laboratory.

## Author Contributions

JM, AG, Y-PC, and KC conceived and planned DAP-seq experiments which were carried out by JM and Y-PC. AG performed quality control analysis and interpretation of sequencing reads. SH performed motif discovery and subsequent bioinformatics analysis and performed EMSAs. SH and AG led initial drafting of the manuscript. KC secured funding to enable this work. All authors contributed to finalizing the manuscript.

## Author Disclaimer

The views expressed in the article do not necessarily represent the views of the DOE or the U.S. Government. The U.S. Government retains and the publisher, by accepting the article for publication, acknowledges that the U.S. Government retains a non-exclusive, paid-up, irrevocable, worldwide license to publish or reproduce the published form of this work, or allow others to do so, for U.S. Government purposes.

## Conflict of Interest

The authors declare that the research was conducted in the absence of any commercial or financial relationships that could be construed as a potential conflict of interest.

## Publisher’s Note

All claims expressed in this article are solely those of the authors and do not necessarily represent those of their affiliated organizations, or those of the publisher, the editors and the reviewers. Any product that may be evaluated in this article, or claim that may be made by its manufacturer, is not guaranteed or endorsed by the publisher.

## Footnotes

1
https://github.com/ibest/HTStream

2
https://bedtools.readthedocs.io/en/latest/

3
https://bedops.readthedocs.io/en/latest/index.html

4
http://pedagogix-tagc.univ-mrs.fr/rsat

## Supplementary Material

Supplementary Figure 1 Scatter plots of cross-experiment correlation between peak fold changes. Nine TFs produced peaks in both the large-scale and targeted repeat DAP-seq experiments. Points are plotted according to the fold change of that peak in the original experiment (horizontal axis) and the repeat experiment (vertical axis). Peaks found in only one experiment are plotted along Y = 0 (large-scale experiment) or X = 0 (repeat experiment). Plots are included for **(A)** Xre_0026, **(B)** AraC_0222, **(C)** TetR_0692, **(D)** BlaI_0696, **(E)** LexA_1449, **(F)** GntR_1482, **(G)** Fur_1691, **(H)** BlaI_1845, and **(I)** Rex_2471.

Supplementary Figure 2 The most enriched DNA-binding sequence motifs among DAP-seq peaks for each TF were consistent across experiments. Motifs derived from peaks identified across different experiments are shown for comparison. The motifs derived from combining all peaks are similar to the motifs from individual experiments **(A)**. No substantial changes were seen in the putative binding sequences detected for GlyR2 (Clo1313_0089) and Rex_2471 from DAP-seq experiments with various amounts of added cell lysate **(B)**.

Supplementary Table 1 RSAT genomes used in this study and the bidirectional best BLAST hit (BBH) orthologs used in this study.

Supplementary Table 2 Gene regulatory networks inferred from DAP-seq and bioinformatic analyses.

Supplementary Table 3 Locus tags and amino acid sequences of proteins tested in the original organism-wide screen.

Supplementary Table 4 All DAP-seq peaks discovered for all TFs.

Supplementary Table 5 Transcription factors used for a targeted DAP-seq experiment in the presence and absence of cell lysate.

Supplementary Table 6 Oligos used for test and negative control EMSAs.

Supplementary File 1 Figures of networks showing the predicted regulon for each TF and a global network encompassing all predicted regulons controlled by the TFs. TFs, in blue diamonds, target regulate genes marked in green and yellow boxes. Genes in yellow boxes are referenced in the main text.
