# Supplementary figures and images for: Genome-Wide Transcription Factor DNA Binding Sites and Gene Regulatory Networks in Clostridium thermocellum
Source: Front Microbiol. 2021 Sep 7;12:695517. doi: 10.3389/fmicb.2021.695517 (PMC8457756; doi:10.3389/fmicb.2021.695517)

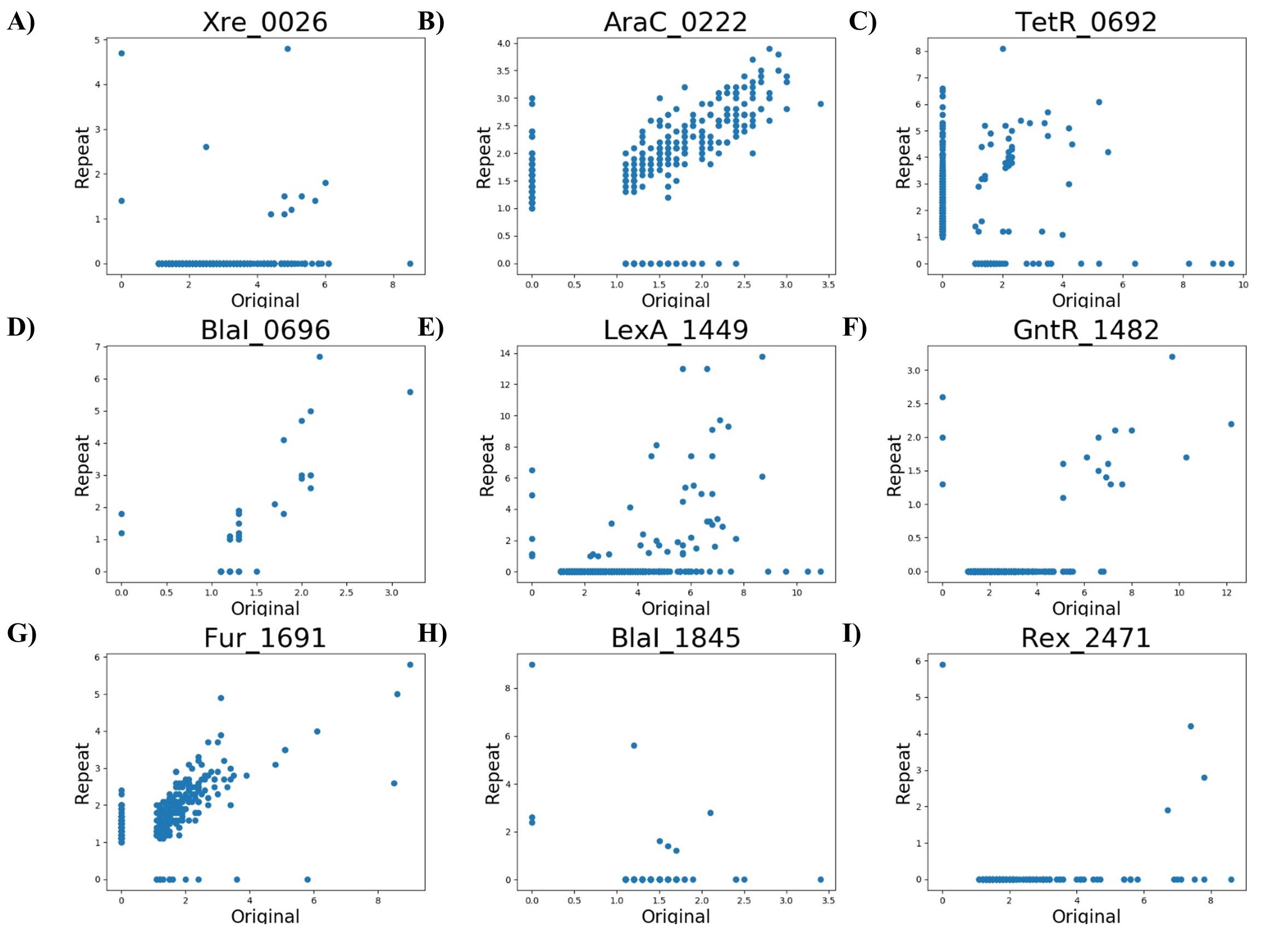

Supplement: Supplementary Figure 1 — Scatter plots of cross-experiment correlation between peak fold changes. Nine TFs produced peaks in both the large-scale and targeted repeat DAP-seq experiments. Points are plotted according to the fold change of that peak in the original experiment (horizontal axis) and the repeat experiment (vertical axis). Peaks found in only one experiment are plotted along Y = 0 (large-scale experiment) or X = 0 (repeat experiment). Plots are included for (A) Xre_0026, (B) AraC_0222, (C) TetR_0692, (D) BlaI_0696, (E) LexA_1449, (F) GntR_1482, (G) Fur_1691, (H) BlaI_1845, and (I) Rex_2471. [file Image_1.JPEG]

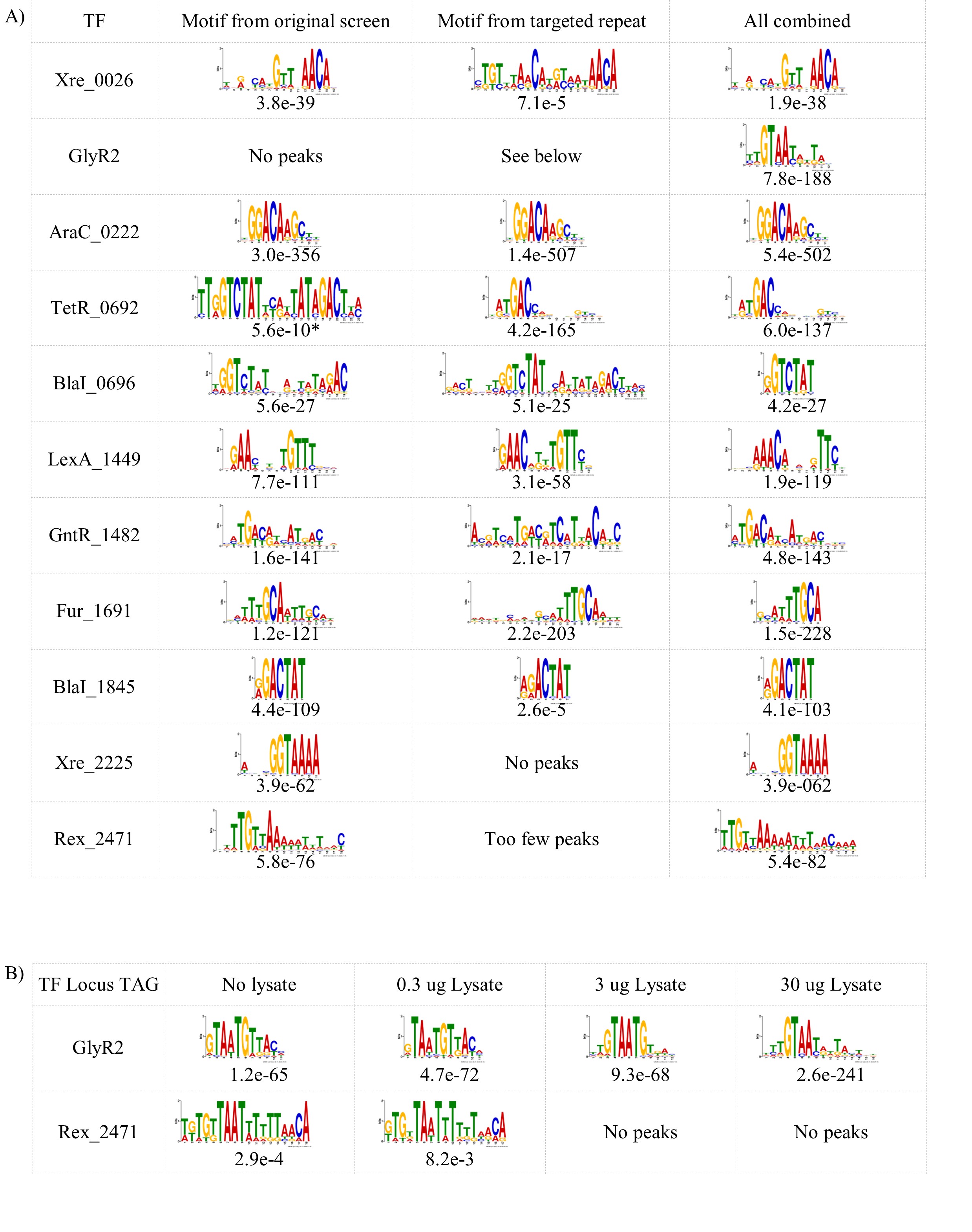

Supplement: Supplementary Figure 2 — The most enriched DNA-binding sequence motifs among DAP-seq peaks for each TF were consistent across experiments. Motifs derived from peaks identified across different experiments are shown for comparison. The motifs derived from combining all peaks are similar to the motifs from individual experiments (A). No substantial changes were seen in the putative binding sequences detected for GlyR2 (Clo1313_0089) and Rex_2471 from DAP-seq experiments with various amounts of added cell lysate (B). [file Image_2.JPEG]
