# Supplementary material for: Genome-Wide Transcription Factor DNA Binding Sites and Gene Regulatory Networks in Clostridium thermocellum
Source: Front Microbiol. 2021 Sep 7;12:695517. doi: 10.3389/fmicb.2021.695517 (PMC8457756; doi:10.3389/fmicb.2021.695517)
Supplement: Supplementary File 1 — Figures of networks showing the predicted regulon for each TF and a global network encompassing all predicted regulons controlled by the TFs. TFs, in blue diamonds, target regulate genes marked in green and yellow boxes. Genes in yellow boxes are referenced in the main text. [file Data_Sheet_1.PDF]

Supplemental Materials for Genome-wide Transcription Factor DNA Binding Sites and Gene Regulatory Networks in Clostridium thermocellum

Files

Figure S1.jpg
Figure S2.jpg
File S1.pdf
Supplemental Tables.xlsx
Supplemental Folder.zip

Contents

Figure S1. Scatter plots of cross-experimental correlatin between peak fold changes.

Peak fold changes are plotted for the original (X-axis) and repeat (Y-axis) experiments. Points along the X=0 and Y=0 represent peaks found in only one of the experiments. Data are available for Xre_0026, AraC_0222, TetR_0692, BlaI_0696, LexA_1449, GntR_1482, Fur_1691, BlaI_1845, and Rex_2471.

Figure S2. The most enriched DNA-binding sequence motifs among DAP-seq peaks for each TF were consistent across experiments.

Motifs derived from peaks identified across different experiments are show for comparison. A) Motifs derived from combining all peaks are similar to the motifs from individual experiments. B) No substantial changes were seen in the putative binding sequences detected for GlyR2 (Clo1313_0089) and Rex_2471 from DAP-seq experiments with various amounts of added cell lysate.

File S1. Figures of networks showing the predicted regulon for each TF and a global network encompassing all predicted regulons controlled by the TFs

TFs are shown in blue diamonds. They regulate genes marked in green and yellow boxes. Genes in yellow boxes are referenced in the main text.

Supplemental Tables.xlsx
Table S1. RSAT genomes used in this study and the bidirectional best BLAST hit (BBH) orthologs used in this study.
Table S2. Gene regulatory networks infered from DAP-seq and bioinformatic analyses
Table S3. Locus tags and amino acid sequences of proteins tested in the original organism-wide screen.
Table S4. All DAP-seq peaks discovered for all TFs.
Table S5. Transcription factors used for a targeted DAP-seq experiment in the presence and absence of cell lysate.
Table S6. Oligos used for test and negative control EMSAs.

Supplemental Folder.zip
A compressed folder containing 3 files for each TF:
1) The 500.fasta contains 500 bp genomic DNA sequences centered around the apex of each peak identified per TF from all DAP-seq experiments.
2) The .fasta.e1600 is the “purged” set of sequences used for input into MEME-Chip analysis.

3) The motif_sequences.fasta file includes the raw sequences that comprise the motifs/sequence logos.

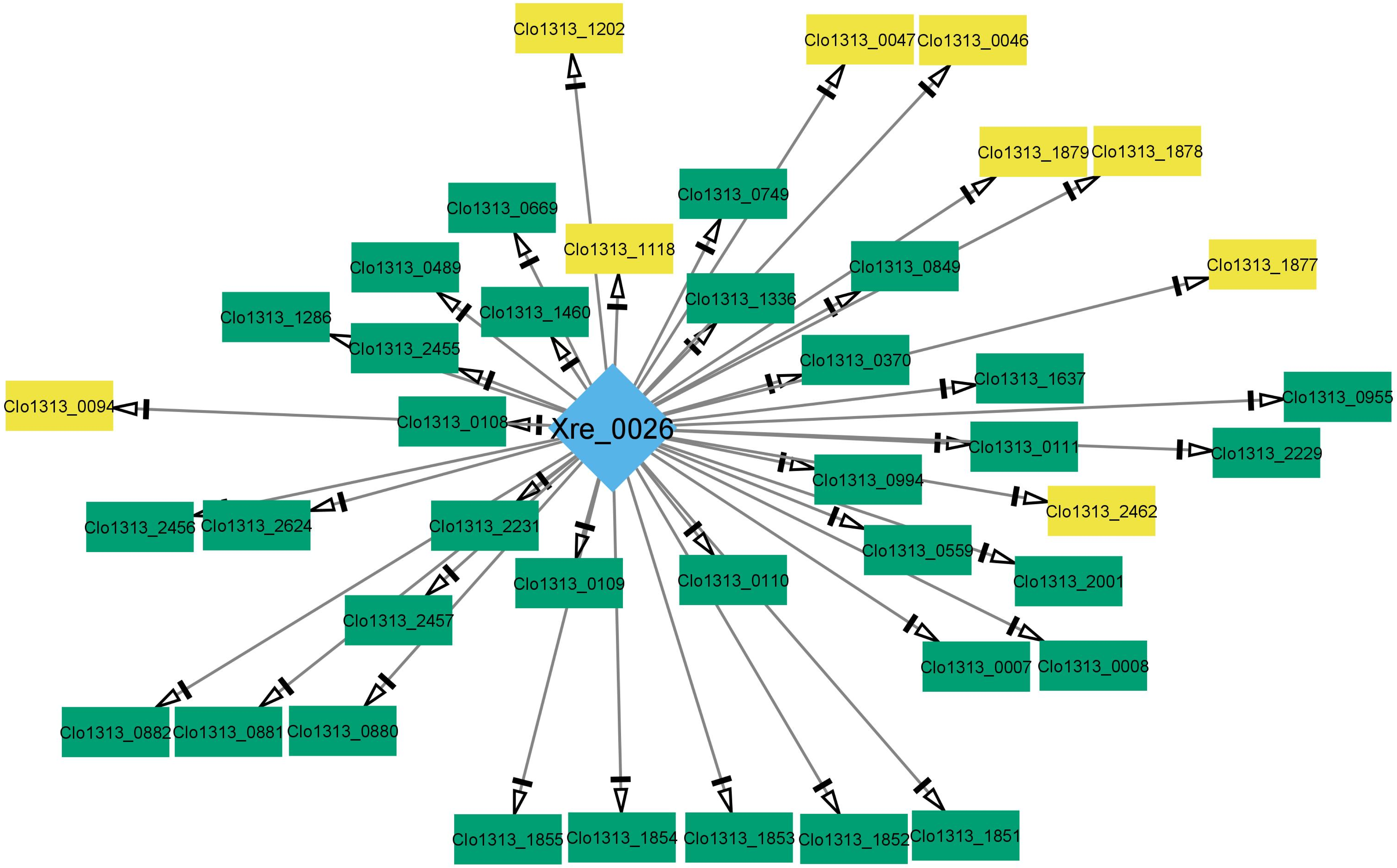
Xre_0026
Clo1313_1202
Clo1313_0047
Clo1313_0046
Clo1313_1879
Clo1313_1878
Clo1313_0749
Clo1313_0669
Clo1313_1118
Clo1313_0489
Clo1313_0849
Clo1313_1877
Clo1313_1336
Clo1313_1460
Clo1313_1286
Clo1313_2455
Clo1313_0370
Clo1313_1637
Clo1313_0955
Clo1313_0094
Clo1313_0108
Clo1313_0111
Clo1313_2229
Clo1313_0994
Clo1313_2462
Clo1313_2456
Clo1313_2624
Clo1313_2231
Clo1313_0559
Clo1313_2001
Clo1313_0109
Clo1313_0110
Clo1313_2457
Clo1313_0007
Clo1313_0008
Clo1313_0882
Clo1313_0881
Clo1313_0880
Clo1313_1855
Clo1313_1854
Clo1313_1853
Clo1313_1852
Clo1313_1851

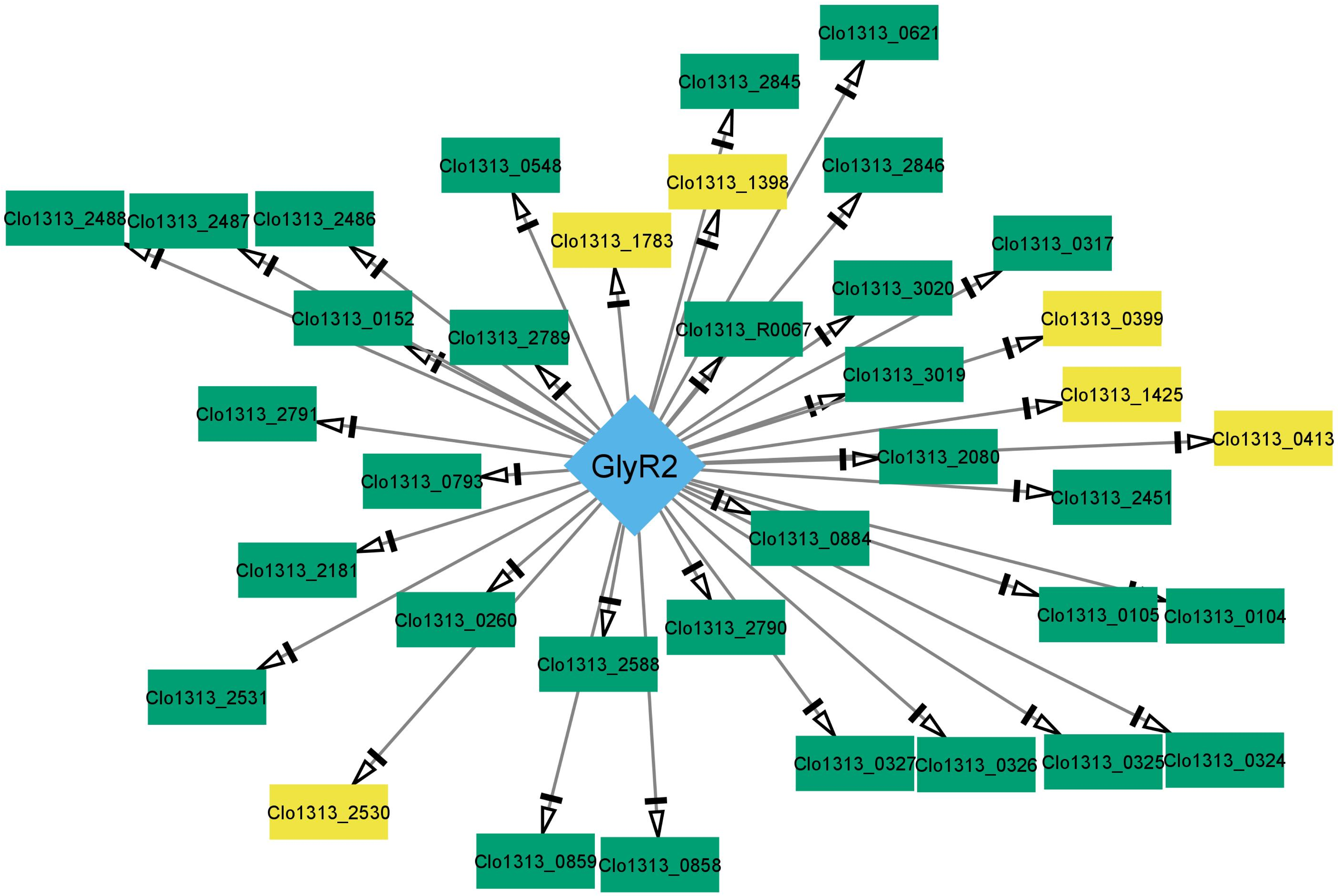
GlyR2
Clo1313_0621
Clo1313_2845
Clo1313_1398
Clo1313_2846
Clo1313_0548
Clo1313_1783
Clo1313_2488
Clo1313_2487
Clo1313_2486
Clo1313_0317
Clo1313_3020
Clo1313_0399
Clo1313_0152
Clo1313_2789
Clo1313_R0067
Clo1313_3019
Clo1313_1425
Clo1313_2791
Clo1313_0413
Clo1313_2080
Clo1313_0793
Clo1313_2451
Clo1313_0884
Clo1313_2181
Clo1313_0105
Clo1313_0104
Clo1313_0260
Clo1313_2790
Clo1313_2588
Clo1313_2531
Clo1313_0327
Clo1313_0326
Clo1313_0325
Clo1313_0324
Clo1313_2530
Clo1313_0859
Clo1313_0858

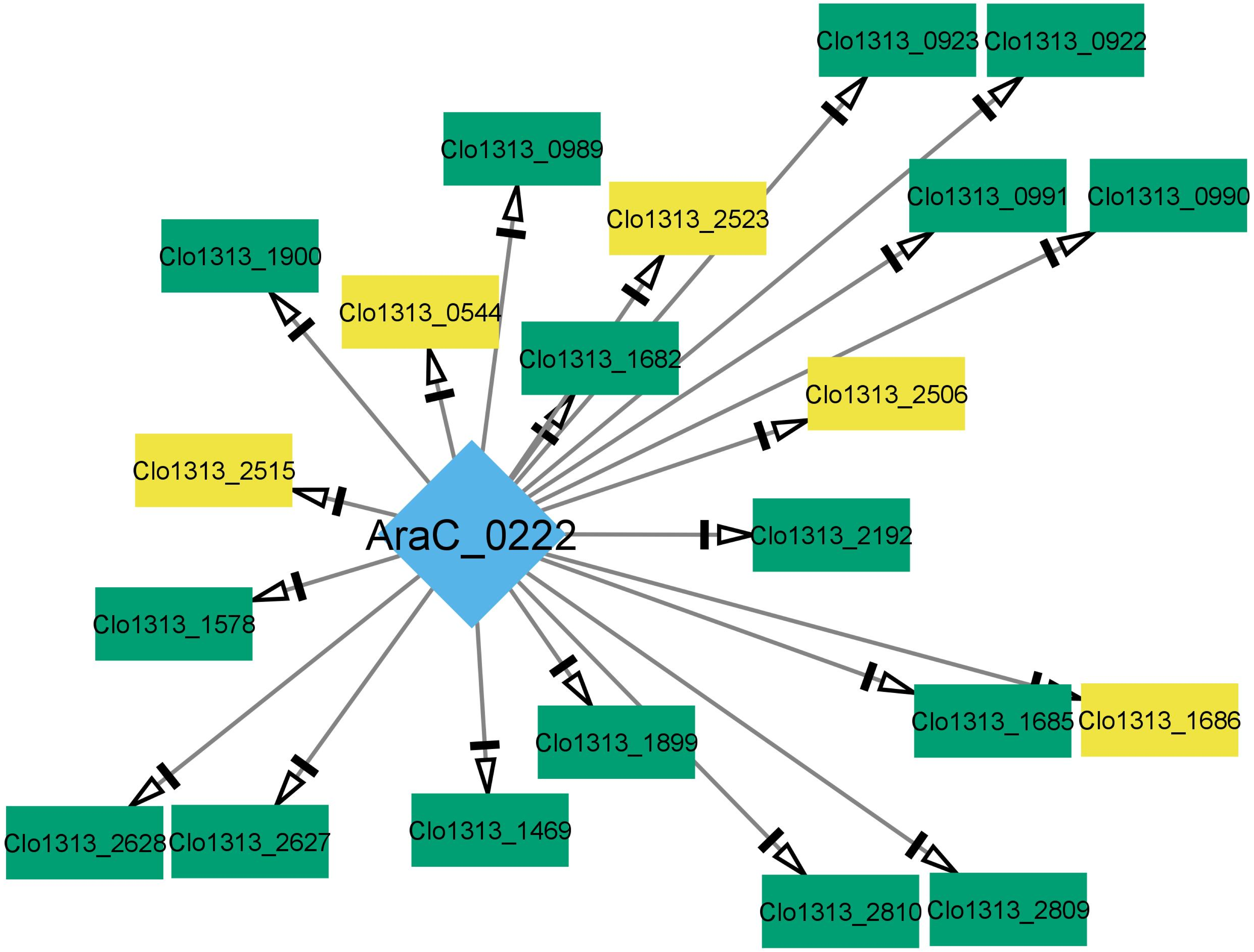

Clo1313_0923
Clo1313_0922
Clo1313_0989
Clo1313_0991
Clo1313_0990
Clo1313_2523
Clo1313_1900
Clo1313_0544
Clo1313_1682
Clo1313_2506
Clo1313_2515
AraC_0222
Clo1313_2192
Clo1313_1578
Clo1313_1685
Clo1313_1686
Clo1313_1899
Clo1313_2628
Clo1313_2627
Clo1313_1469
Clo1313_2810
Clo1313_2809

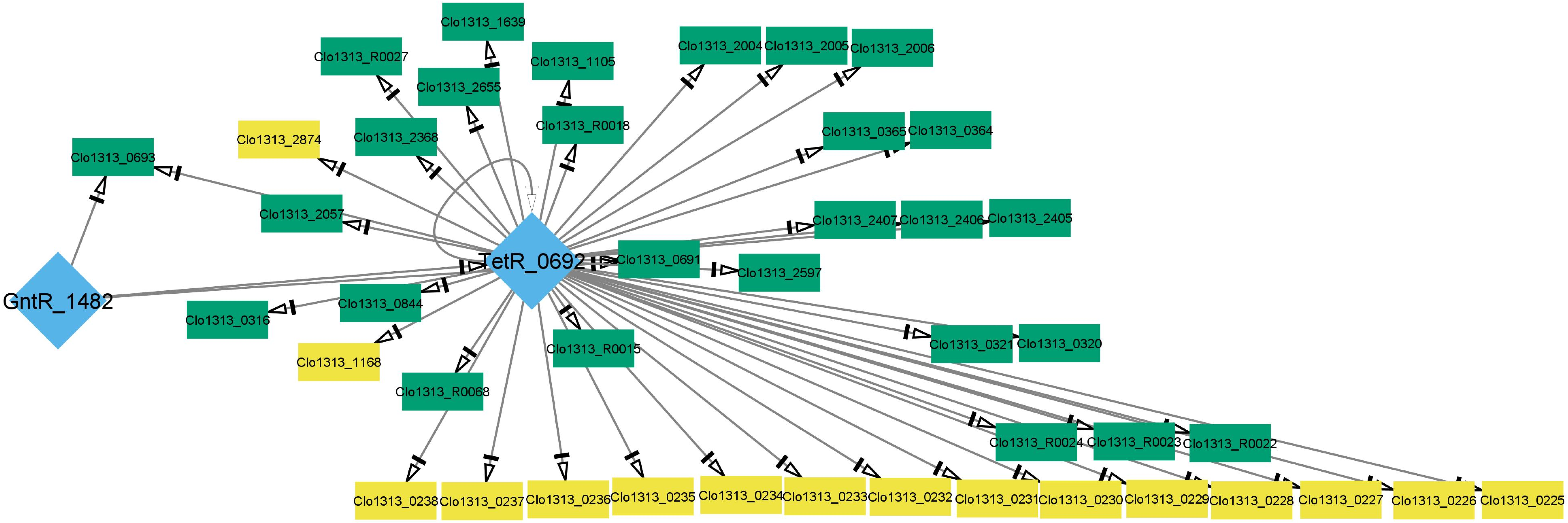

GntR_1482
TetR_0692
Clo1313_0693
Clo1313_2057
Clo1313_2874
Clo1313_2368
Clo1313_R0027
Clo1313_2655
Clo1313_1639
Clo1313_1105
Clo1313_R0018
Clo1313_2004
Clo1313_2005
Clo1313_2006
Clo1313_0365
Clo1313_0364
Clo1313_2407
Clo1313_2406
Clo1313_2405
Clo1313_0691
Clo1313_2597
Clo1313_0844
Clo1313_0316
Clo1313_1168
Clo1313_R0068
Clo1313_R0015
Clo1313_0321
Clo1313_0320
Clo1313_R0024
Clo1313_R0023
Clo1313_R0022
Clo1313_0238
Clo1313_0237
Clo1313_0236
Clo1313_0235
Clo1313_0234
Clo1313_0233
Clo1313_0232
Clo1313_0231
Clo1313_0230
Clo1313_0229
Clo1313_0228
Clo1313_0227
Clo1313_0226
Clo1313_0225

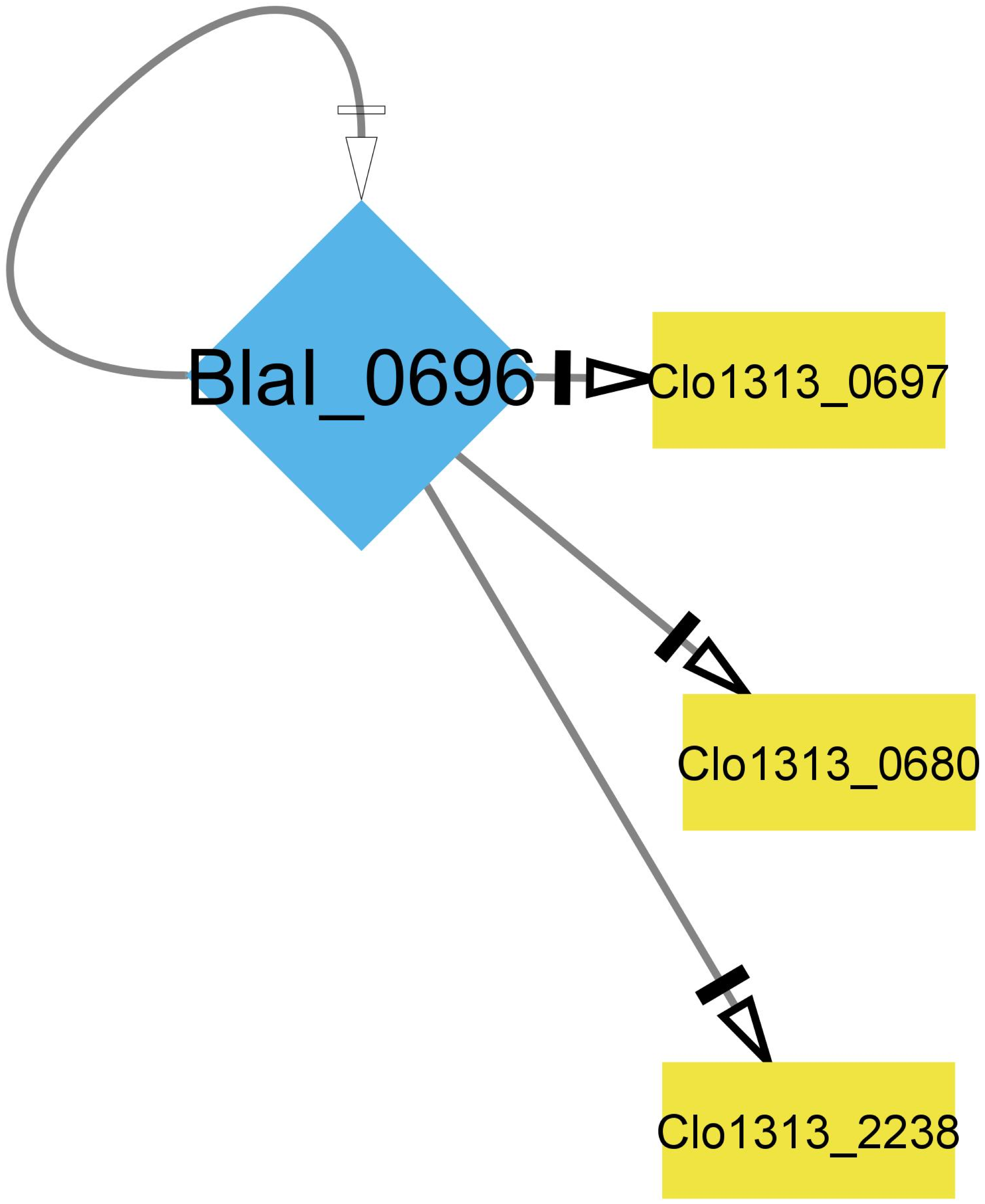
BlaI_0696
Clo1313_0697
Clo1313_0680
Clo1313_2238

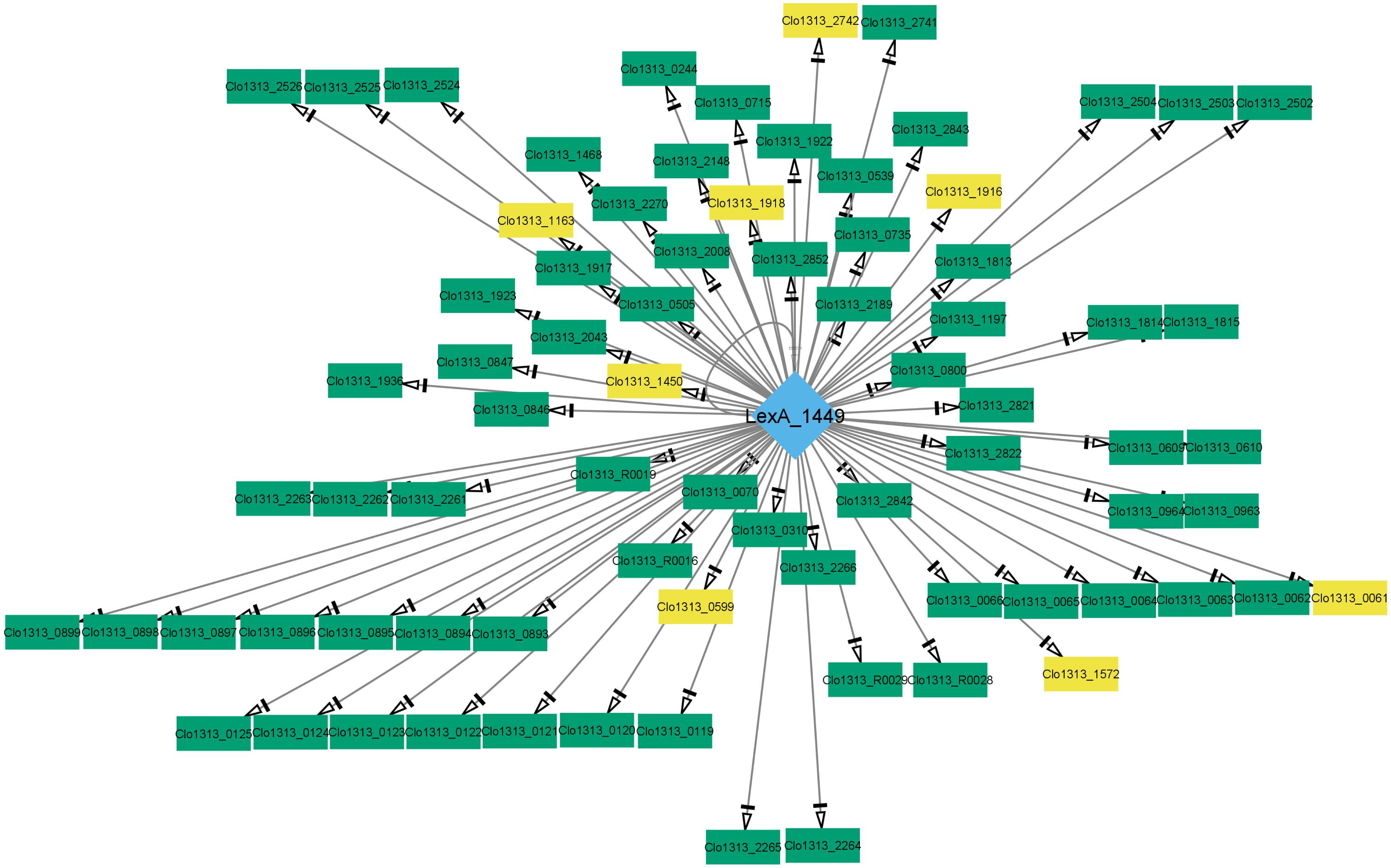
Clo1313_2742
Clo1313_2741
Clo1313_0244
Clo1313_2526
Clo1313_2525
Clo1313_2524
Clo1313_0715
Clo1313_2504
Clo1313_2503
Clo1313_2502
Clo1313_1922
Clo1313_2843
Clo1313_1468
Clo1313_2148
Clo1313_0539
Clo1313_1916
Clo1313_2270
Clo1313_1918
Clo1313_1163
Clo1313_0735
Clo1313_2008
Clo1313_2852
Clo1313_1813
Clo1313_1917
Clo1313_1923
Clo1313_0505
Clo1313_2189
Clo1313_1197
Clo1313_1814
Clo1313_1815
Clo1313_2043
Clo1313_0800
Clo1313_0847
Clo1313_1450
Clo1313_1936
Clo1313_2821
Clo1313_0846
LexA_1449
Clo1313_0609
Clo1313_0610
Clo1313_2822
Clo1313_R0019
Clo1313_2263
Clo1313_2262
Clo1313_2261
Clo1313_0070
Clo1313_2842
Clo1313_0964
Clo1313_0963
Clo1313_0310
Clo1313_R0016
Clo1313_2266
Clo1313_0066
Clo1313_0065
Clo1313_0064
Clo1313_0063
Clo1313_0062
Clo1313_0061
Clo1313_0599
Clo1313_0899
Clo1313_0898
Clo1313_0897
Clo1313_0896
Clo1313_0895
Clo1313_0894
Clo1313_0893
Clo1313_R0029
Clo1313_R0028
Clo1313_1572
Clo1313_0125
Clo1313_0124
Clo1313_0123
Clo1313_0122
Clo1313_0121
Clo1313_0120
Clo1313_0119
Clo1313_2265
Clo1313_2264

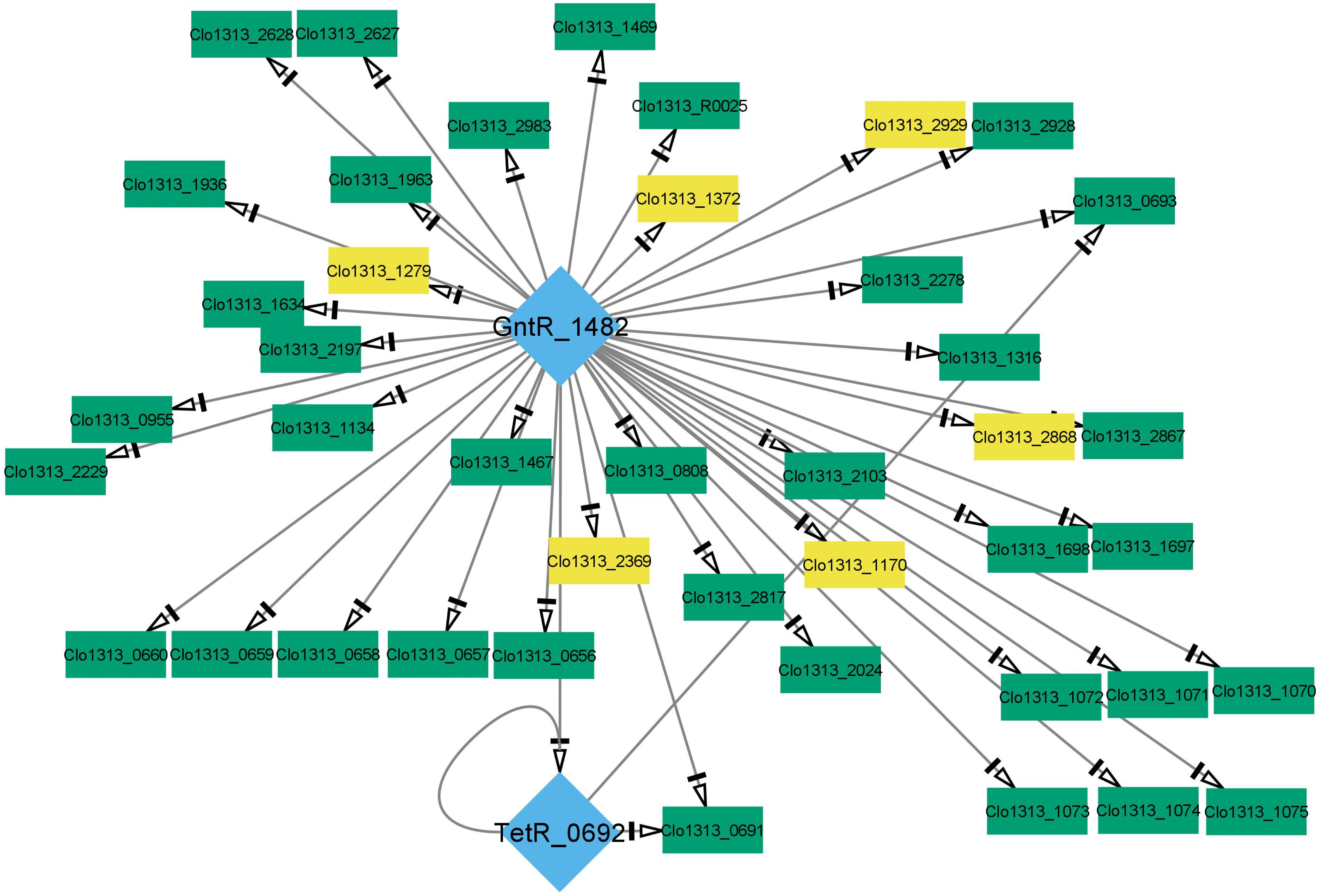
Clo1313_2628
Clo1313_2627
Clo1313_1469
Clo1313_R0025
Clo1313_2983
Clo1313_2929
Clo1313_2928
Clo1313_1936
Clo1313_1963
Clo1313_1372
Clo1313_0693
Clo1313_1279
Clo1313_2278
Clo1313_1634
GntR_1482
Clo1313_2197
Clo1313_1316
Clo1313_0955
Clo1313_1134
Clo1313_2868
Clo1313_2867
Clo1313_2229
Clo1313_1467
Clo1313_0808
Clo1313_2103
Clo1313_1698
Clo1313_1697
Clo1313_2369
Clo1313_1170
Clo1313_2817
Clo1313_0660
Clo1313_0659
Clo1313_0658
Clo1313_0657
Clo1313_0656
Clo1313_2024
Clo1313_1072
Clo1313_1071
Clo1313_1070
Clo1313_1073
Clo1313_1074
Clo1313_1075
TetR_0692
Clo1313_0691

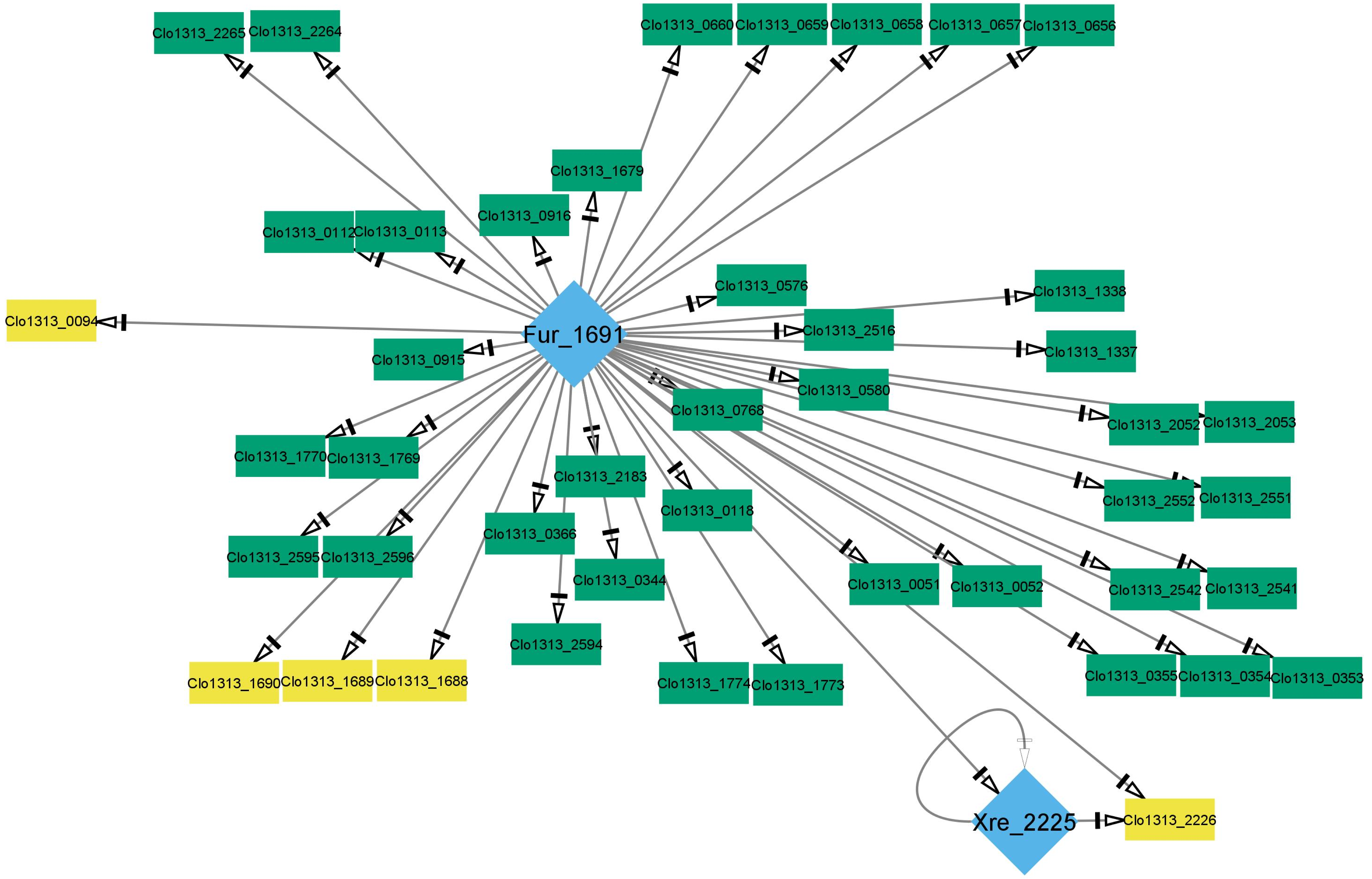

Fur_1691
Xre_2225
Clo1313_2265
Clo1313_2264
Clo1313_0660
Clo1313_0659
Clo1313_0658
Clo1313_0657
Clo1313_0656
Clo1313_1679
Clo1313_0916
Clo1313_0112
Clo1313_0113
Clo1313_0576
Clo1313_1338
Clo1313_0094
Clo1313_2516
Clo1313_1337
Clo1313_0915
Clo1313_0580
Clo1313_0768
Clo1313_2052
Clo1313_2053
Clo1313_1770
Clo1313_1769
Clo1313_2183
Clo1313_2552
Clo1313_2551
Clo1313_0118
Clo1313_2595
Clo1313_2596
Clo1313_0366
Clo1313_0344
Clo1313_0051
Clo1313_0052
Clo1313_2542
Clo1313_2541
Clo1313_2594
Clo1313_1690
Clo1313_1689
Clo1313_1688
Clo1313_1774
Clo1313_1773
Clo1313_0355
Clo1313_0354
Clo1313_0353
Clo1313_2226

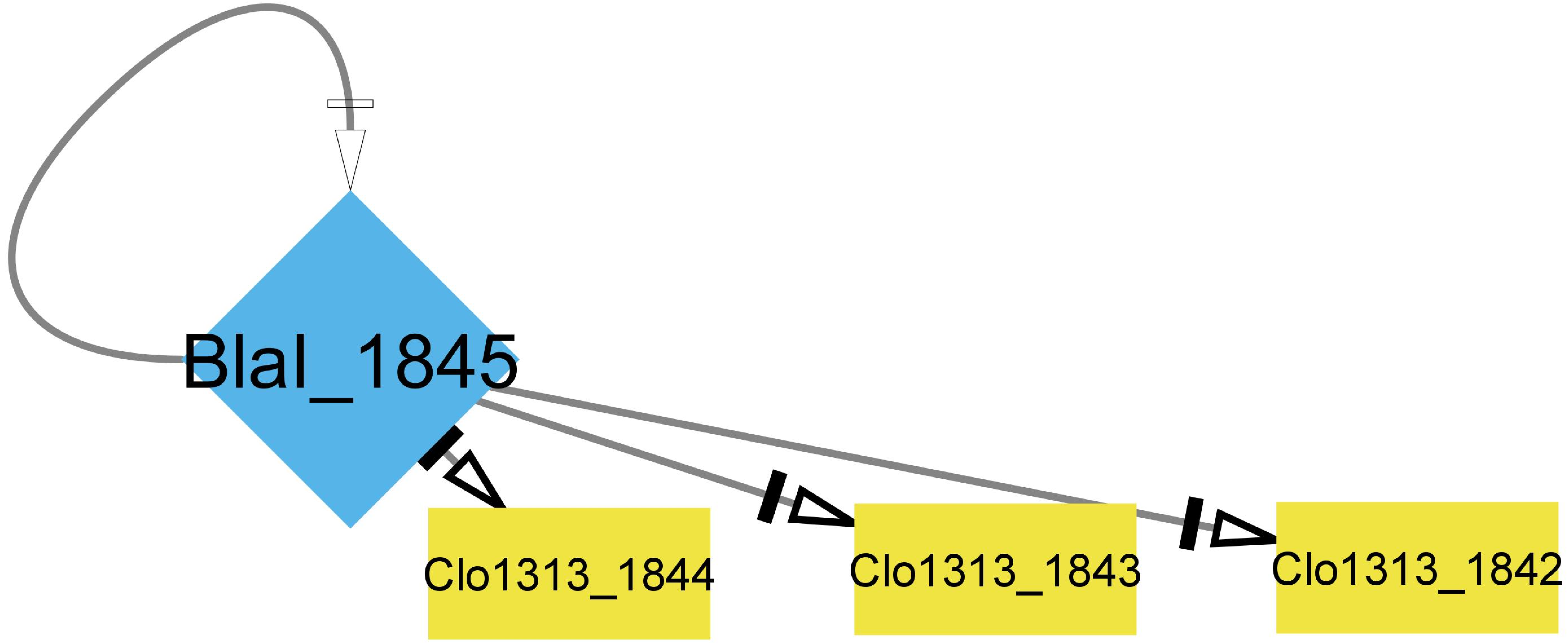
BlaI_1845
Clo1313_1844
Clo1313_1843
Clo1313_1842

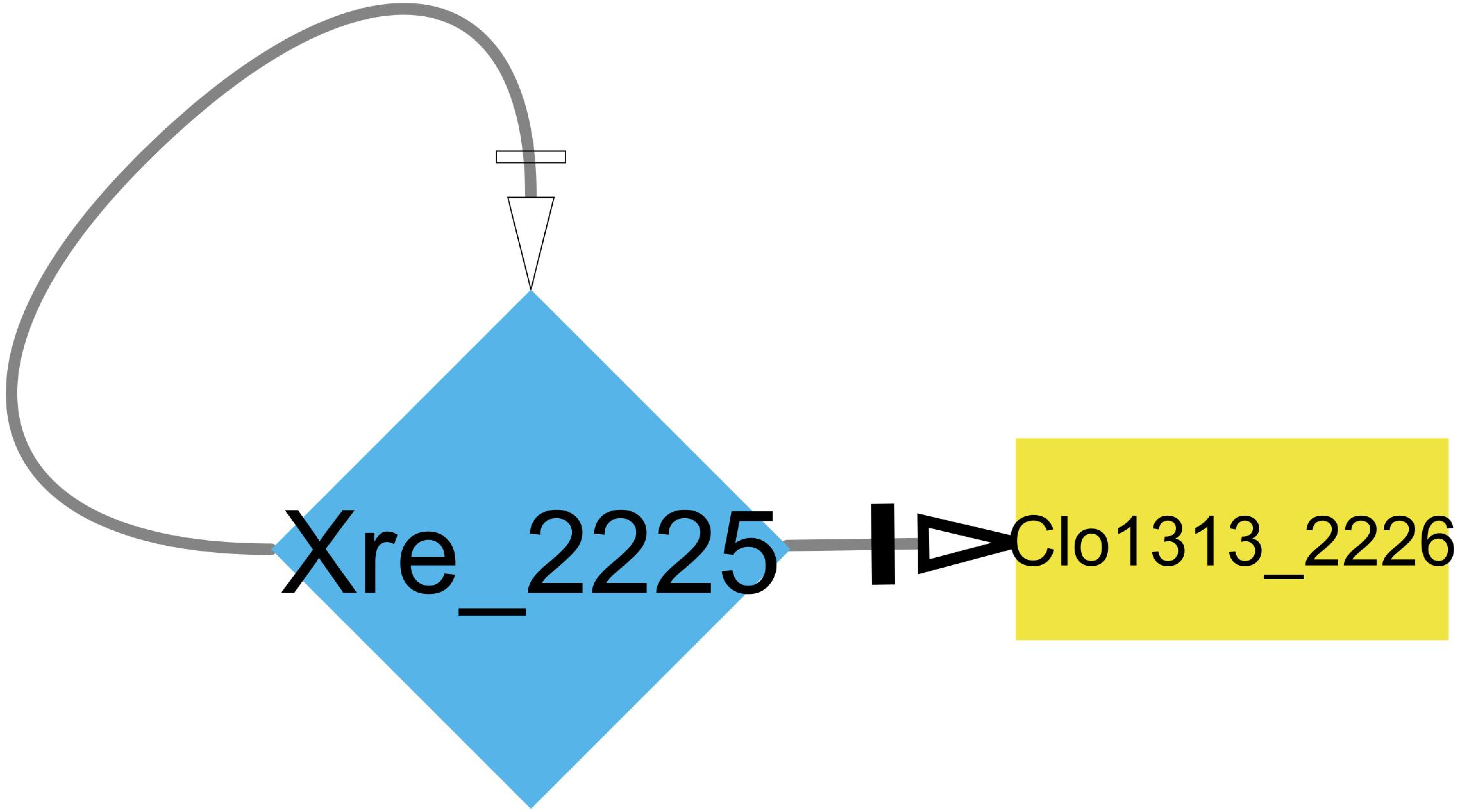
Xre_2225
Clo1313_2226

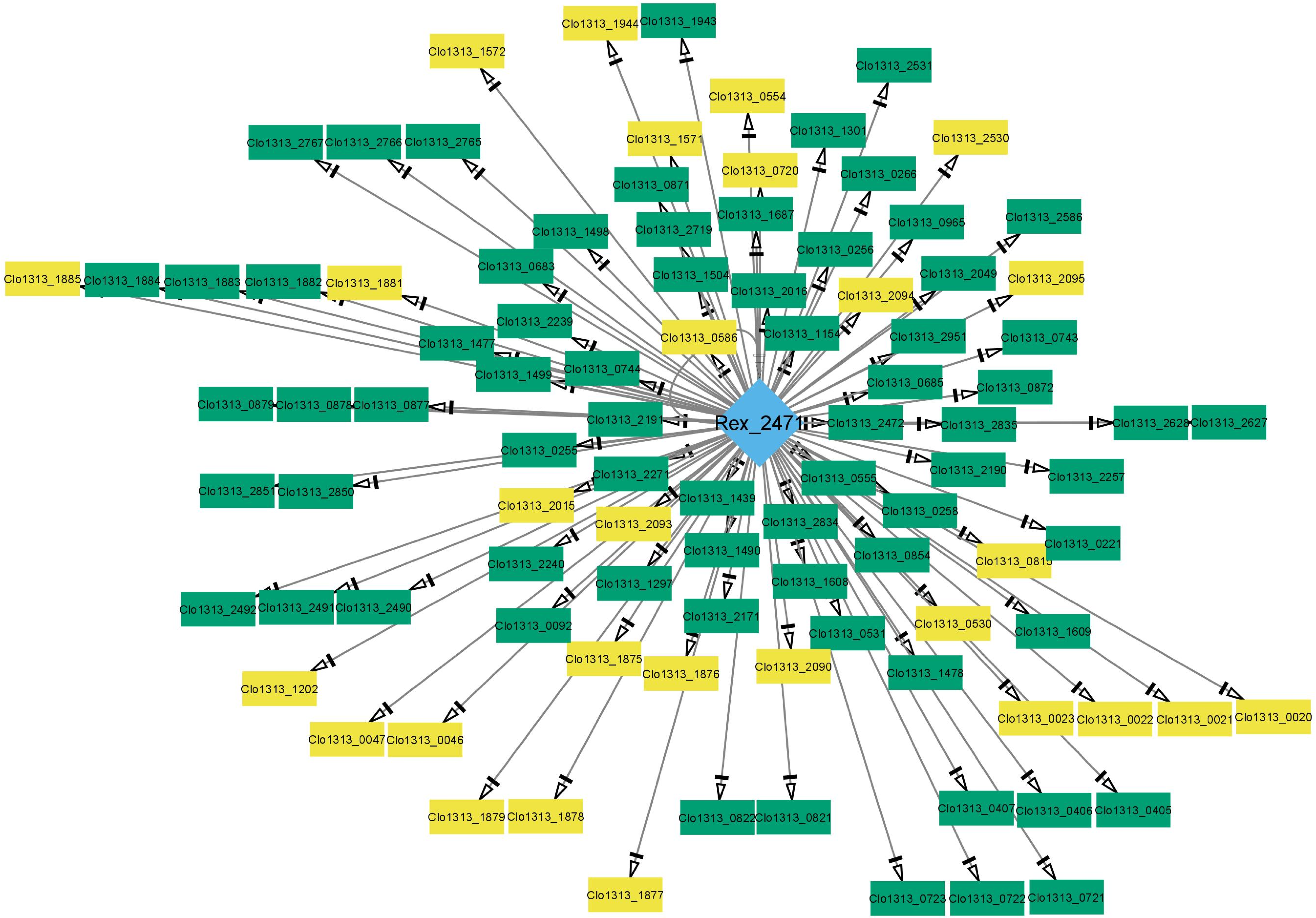
Clo1313_1944
Clo1313_1943
Clo1313_1572
Clo1313_2531
Clo1313_0554
Clo1313_1301
Clo1313_2767
Clo1313_2766
Clo1313_2765
Clo1313_1571
Clo1313_2530
Clo1313_0720
Clo1313_0871
Clo1313_0266
Clo1313_1687
Clo1313_2586
Clo1313_1498
Clo1313_2719
Clo1313_0965
Clo1313_0256
Clo1313_0683
Clo1313_2049
Clo1313_2095
Clo1313_1885
Clo1313_1884
Clo1313_1883
Clo1313_1882
Clo1313_1881
Clo1313_1504
Clo1313_2016
Clo1313_2094
Clo1313_2239
Clo1313_1154
Clo1313_2951
Clo1313_0743
Clo1313_1477
Clo1313_0586
Clo1313_0744
Clo1313_1499
Clo1313_0685
Clo1313_0872
Clo1313_0879
Clo1313_0878
Clo1313_0877
Rex_2471
Clo1313_2472
Clo1313_2835
Clo1313_2628
Clo1313_2627
Clo1313_0255
Clo1313_2190
Clo1313_2257
Clo1313_2851
Clo1313_2850
Clo1313_2271
Clo1313_0555
Clo1313_2015
Clo1313_1439
Clo1313_0258
Clo1313_2834
Clo1313_2093
Clo1313_0221
Clo1313_0854
Clo1313_2240
Clo1313_1490
Clo1313_1608
Clo1313_1297
Clo1313_2492
Clo1313_2491
Clo1313_2490
Clo1313_0530
Clo1313_1609
Clo1313_0092
Clo1313_2171
Clo1313_0531
Clo1313_1875
Clo1313_1876
Clo1313_2090
Clo1313_1478
Clo1313_1202
Clo1313_0023
Clo1313_0022
Clo1313_0021
Clo1313_0020
Clo1313_0047
Clo1313_0046
Clo1313_0407
Clo1313_0406
Clo1313_0405
Clo1313_1879
Clo1313_1878
Clo1313_0822
Clo1313_0821
Clo1313_1877
Clo1313_0723
Clo1313_0722
Clo1313_0721
