# Supplementary material for: Genome-Wide Transcription Factor DNA Binding Sites and Gene Regulatory Networks in Clostridium thermocellum
Source: Front Microbiol. 2021 Sep 7;12:695517. doi: 10.3389/fmicb.2021.695517 (PMC8457756; doi:10.3389/fmicb.2021.695517)
Supplement: Supplementary file 5 [file Data_Sheet_2.ZIP › Supplemental folder/A Table of Contents.docx]

Supplemental Materials for Genome-wide Transcription Factor DNA Binding Sites and Gene Regulatory Networks in Clostridium thermocellum

**Files**

Figure S1.jpg

Figure S2.jpg

File S1.pdf

Supplemental Tables.xlsx

Supplemental Folder.zip

**Contents**

**Figure S1.** Scatter plots of cross-experimental correlatin between peak fold changes.

Peak fold changes are plotted for the original (X-axis) and repeat (Y-axis) experiments. Points along the X=0 and Y=0 represent peaks found in only one of the experiments. Data are available for Xre_0026, AraC_0222, TetR_0692, BlaI_0696, LexA_1449, GntR_1482, Fur_1691, BlaI_1845, and Rex_2471.

**Figure S2.** The most enriched DNA-binding sequence motifs among DAP-seq peaks for each TF were consistent across experiments.

Motifs derived from peaks identified across different experiments are show for comparison. A) Motifs derived from combining all peaks are similar to the motifs from individual experiments. B) No substantial changes were seen in the putative binding sequences detected for GlyR2 (Clo1313_0089) and Rex_2471 from DAP-seq experiments with various amounts of added cell lysate.

**File S1.** Figures of networks showing the predicted regulon for each TF and a global network encompassing all predicted regulons controlled by the TFs

TFs are shown in blue diamonds. They regulate genes marked in green and yellow boxes. Genes in yellow boxes are referenced in the main text.

**Supplemental Tables.xlsx**

**Table S1.** RSAT genomes used in this study and the bidirectional best BLAST hit (BBH) orthologs used in this study.

**Table S2.** Gene regulatory networks infered from DAP-seq and bioinformatic analyses

**Table S3.** Locus tags and amino acid sequences of proteins tested in the original organism-wide screen.

**Table S4.** All DAP-seq peaks discovered for all TFs.

**Table S5.** Transcription factors used for a targeted DAP-seq experiment in the presence and absence of cell lysate.

**Table S6.** Oligos used for test and negative control EMSAs.

**Supplemental Folder.zip**

A compressed folder containing 3 files for each TF:

1) The 500.fasta contains 500 bp genomic DNA sequences centered around the apex of each peak identified per TF from all DAP-seq experiments.

2) The .fasta.e1600 is the “purged” set of sequences used for input into MEME-Chip analysis.

3) The motif_sequences.fasta file includes the raw sequences that comprise the motifs/sequence logos.
